# Patient and donor antibody profiles in early COVID-19 convalescent plasma therapy in the COnV-ert trial

**DOI:** 10.3389/fimmu.2025.1647488

**Published:** 2025-09-25

**Authors:** Andrea Alemany, Dan Ouchi, Edwards Pradenas, Ruth Aguilar, Marta Vidal, Alfons Jimenez, Pere Millat-Martinez, Marc Corbacho-Monné, Clara Suñer, Quique Bassat, Bàrbara Baro, Gemma Moncunill, Oriol Mitjà, COnV-ert Group of Authors, Julià Blanco, Carlota Dobaño

**Affiliations:** ^1^ Fight Infectious Diseases Foundation, Badalona, Spain; ^2^ Hospital Universitari Germans Trias i Pujol, Badalona, Spain; ^3^ ISGlobal, Barcelona, Spain; ^4^ Facultat de Medicina i Ciències de la Salut, Universitat de Barcelona (UB), Barcelona, Spain; ^5^ IrsiCaixa, Badalona, Spain; ^6^ Parc Taulí Hospital Universitari, Institut d’Investigació i Innovació Parc Taulí (I3PT-CERCA), Sabadell, Spain; ^7^ Consorcio de Investigación Biomédica en Red de Epidemiología y Salud Pública (CIBERESP), Instituto de Salud Carlos III, Madrid, Spain; ^8^ ICREA, Barcelona, Spain; ^9^ Pediatrics Department, Hospital Sant Joan de Déu, Universitat de Barcelona, Esplugues, Barcelona, Spain; ^10^ Centro de Investigação em Saúde de Manhiça (CISM), Maputo, Mozambique; ^11^ CIBER Enfermedades Infecciosas (CIBERINFEC), Barcelona, Spain; ^12^ Universitat de Vic - Universitat Central de Catalunya (UVIC-UCC), Vic, Spain; ^13^ Germans Trias i Pujol Research Institute (IGTP), Badalona, Spain

**Keywords:** high-titer convalescent plasma, COVID-19, antibody immune response, methylene blue, immunology & infectious diseases

## Abstract

**Introduction:**

The negative efficacy results of coronavirus disease 2019 (COVID-19) convalescent plasma (CCP) as early treatment in the COnV-ert trial have been attributed to the use of methylene blue (MB). We characterized immune responses after MB-treated CCP infusion and the impact of MB on antibodies of the infused CCP units.

**Methods:**

We measured antibody isotypes (IgG, IgM, and IgA) and IgG subclasses (IgG1, IgG2, IgG3, and IgG4) against SARS-CoV-2 nucleocapsid and spike (S) antigens, neutralizing antibody titers, and IgG avidity in 128 participants of the COnV-ert trial 7 and 60 days after infusion and in paired CCP units before and after MB treatment.

**Results:**

Treatment with CCP significantly increased the levels of IgG and IgG1 to receptor-binding domain (RBD) and S, IgG3 to S and S2, and IgG avidity in recipients 7 days after infusion, without an increase in IgA, IgM, IgG2, IgG4, or neutralization. At day 7 post-infusion, recipients exhibited lower IgG, all IgG subclasses, and avidity; higher IgA and IgM; and comparable neutralization relative to paired CCP units. MB was associated with a significant decrease in cytophilic subclasses IgG1 and IgG3 to S and S2, and IgA to RBD, S and S2 in CCP units, without a reduction in neutralization titer and with a modest increase in IgG2 to RBD and S.

**Discussion:**

Our study shows a modest impact of a single intravenous infusion of MB-treated high-titer CCP on circulating antibody levels compared to those generated by the host by day 7 and an adverse effect of MB on IgG1 and IgG3, which are essential for effector functions.

**Clinical trial registration:**

https://www.clinicaltrials.gov/, identifier NCT04621123.

## Introduction

COVID-19 convalescent plasma (CCP) has been deployed globally during the coronavirus disease 2019 (COVID-19) pandemic in attempts to reduce disease progression and mortality and remains a valuable option for selected patients unable to tolerate or relapsing after antiviral treatments (1, 2). Several trials have investigated the use of CCP for both inpatients and outpatients, as well as immunocompromised individuals, yielding mixed results and efficacy notably driven by the early administration of the product with high antibody titers (3–9).

Five well-designed randomized controlled trials (RCTs) have evaluated the efficacy of CCP as early treatment in outpatients, prior to the emergence of the Omicron variants and administering CCP from non-vaccinated donors (10–14). Two of these trials, conducted in Argentina (CCP-Argentina) (12) and the USA (CSSC-004) (14), showed a relative reduction of 30% and 50% of the risk of disease progression and hospitalization, respectively. Conversely, CCP was not associated with a lower likelihood of disease progression in the other three trials conducted in the USA (C3PO) (11), Spain (COnV-ert) (10), and the Netherlands (COV-Early) (13). A meta-analysis including individual participant data from all five RCTs showed a 30% relative risk reduction (RRR) for all-cause hospitalization, with the greatest reduction (50% RRR) in with earlier administration (≤5 days after symptom onset) of CCP with higher antibody titers (above the median titer for each RCT) (3).

These inconsistent efficacy results across trials may be attributed to significant variability in the levels and functionality of SARS-CoV-2 antibodies in CCP units, the methods used for testing, and the pathogen inactivation techniques applied (15). Methylene blue (MB), a pathogen reduction method approved by the Food and Drug Administration (FDA) and used to treat the CCP administered in the COnV-ert trial, has been hypothesized to contribute to the observed lack of efficacy (16, 17). However, evidence on the impact of MB treatment and other inactivation methods on immunoglobulin levels and functions remains limited. While few studies suggest that MB preserves the neutralizing activity of CCP (18, 19), the overall effects of these technologies, mainly on Fc-dependent antibody functions, are not well understood, with existing data presenting conflicting findings on the extent of antibody impairment (16, 17, 20).

The aims of this current study of the COnV-ert trial were 1) to characterize the antibody immune responses in COVID-19 outpatients 7 and 60 days after early treatment with a single intravenous infusion of 250–300 mL of MB-treated high-titer CCP (from non-vaccinated donors) and 2) to characterize the anti-SARS-CoV-2 antibody profile in the infused high-titer CCP before and after treatment with MB.

## Methods

### Study design and approval

The current study is a secondary analysis of the COnV-ert trial, a multicenter, double-blind, randomized, placebo-controlled trial assessing the efficacy of early treatment with high-titer MB-treated CCP to reduce the risk of hospitalization up to day 28 and the viral load at day 7 (10). The trial was conducted between November 10, 2020, and July 28, 2021, at four health-care centers in Catalonia, Spain (Hospital Universitari Germans Trias i Pujol, Hospital Universitari de Bellvitge, CUAP Manresa, and Hospital Comarcal de Sant Bernabé). The COnV-ert trial and the current analysis were approved by the Ethics Committee at Hospital Germans Trias i Pujol (number PI 20-313) and the institutional review boards of all participating centers. All study participants and plasma donors provided written informed consent.

### Study population

Eligible participants for the COnV-ert study were aged 50 years or older, regardless of other risk factors for severe disease, and with confirmed SARS-CoV-2 infection within 5 days from enrolment. All patients had to be non-hospitalized and present with mild-to-moderate COVID-19 with symptom onset of no more than 7 days. Exclusion criteria included severe COVID-19, hospitalization for any cause, having received a COVID-19 vaccine, contraindications to the investigational product, increased thrombotic risk, and being pregnant or breastfeeding. All participants were randomized 1:1 to receive a single intravenous infusion of either 250–300 mL of high-titer MB-treated CCP (from non-vaccinated donors) or 250 mL of sterile 0.9% saline solution as a placebo. A total of 376 participants were enrolled in the COnV-ert study; and the trial showed no significant differences in hospitalization up to day 28 or change in viral load from baseline to day 7 between the two groups. There was also no evidence of benefit in the clinical or virological outcomes in the sensitivity analysis according to the serostatus of participants at baseline or neutralizing activity of infused CCP.

For this study, we included the first consecutive 135 participants enrolled in the COnV-ert trial from the main study center (i.e., Hospital Universitari Germans Trias i Pujol), which recruited 275 of the 376 total participants included in the study (73.1%), for assessment and comparison of antibody immune responses between treatment groups. We excluded participants without available samples at baseline, day 7, and/or day 60 after infusion from the analyses, resulting in a final analysis population of 128 participants.

### COVID-19 convalescent plasma

All CCP units infused in the COnV-ert study were sourced from a central blood bank (*Banc de Sang i Teixits de Catalunya*, Barcelona) and were selected after screening for high anti-SARS-CoV-2 IgG titers measured using ELISA (EUROIMMUN ratio ≥6), according to the FDA guidelines. CCP was obtained via plasmapheresis from donors with a prior diagnosis of COVID-19 documented by a positive RT-PCR or SARS-CoV-2 antigen test and after a deferral period of at least 14 days after symptom resolution. All donors had to be at least 18 years old and meet standard criteria for blood donation. CCP units were inactivated with methylene blue before transfusion, following standard Spanish blood and tissue bank procedures.

We selected every paired CCP unit corresponding to the infused participants who were included in the current study. We included two matched CCP samples: 1) one sample from a stored biospecimen from the donor (i.e., before MB treatment) and 2) a sample from the CCP units infused (i.e., after MB treatment).

### Neutralizing antibody assay testing

Neutralizing antibody titers were analyzed at *IrsiCaixa* laboratory using a pseudovirus-based neutralization assay, with HIV reporter pseudoviruses expressing SARS-CoV-2 spike protein from the ancestral strain [Wuhan-Hu-1 (WH1)] and carrying the Luciferase gene, as previously described (21, 22). All samples were retrospectively analyzed using plasma samples from participants and CCP units (donors) stored at −80 °C. Briefly, neutralization assays were performed in duplicate in Nunc 96-well cell culture plates (Thermo Fisher Scientific, Waltham, Massachusetts, United States), and 200 TCID50 of pseudovirus were preincubated with threefold serial dilutions (1/60–1/14,580) of heat-inactivated (56°C for 30 min) plasma samples for 1 h at 37°C. Then, 1 × 10^4^ HEK293T/hACE2 cells treated with DEAE-Dextran (Sigma-Aldrich, Saint Louis, United States) were added. Results were read after 48 h using the EnSight Multimode Plate Reader and BriteLite Plus Luciferase reagent (PerkinElmer, Barcelona, Spain). The neutralization capacity of the plasma samples was calculated by comparing the experimental relative light units (RLUs) calculated from infected cells treated with each plasma to the max RLUs (maximal infectivity calculated from untreated infected cells) and min RLUs (minimal infectivity calculated from uninfected cells) and expressed as percent neutralization: %Neutralization = (RLUmax − RLUexperimental)/(RLUmax − RLUmin) * 100. ID_50_ (reciprocal dilution inhibiting 50% of the infection) was calculated by plotting and fitting the log of plasma dilution vs. normalized response to a four-parameter equation in Prism 9 (GraphPad Software).

### Antibody isotypes and subclasses quantification and antibody avidity

Multiplex immunoassays were performed to measure the levels of anti-SARS-CoV-2 antibody isotypes (IgG, IgM, and IgA) and subclasses (IgG1, IgG2, IgG3, and IgG4) against a panel of five antigens—nucleocapsid (N) C-terminal region (CT), N full protein (FL), receptor-binding domain (RBD), spike full protein (S), and S2 fragment (S2) —at the Immune Response and Biomarker Core Facility at ISGlobal following a previously described protocol (23). NFL and NCT were expressed in *Escherichia coli* and His tag-purified at ISGlobal (24). The ancestral S and RBD proteins were fused with C-terminal 6xHis and StrepTag sequences and purified from the supernatant of lentiviral-transduced CHO-S cells cultured under a fed-batch (25). S2 was purchased from Sino Biological. Plasma samples from participants and CCP units (donors) stored at −80 °C were retrospectively analyzed together with 129 prepandemic plasma samples as negative controls to stablish the seropositive cutoff. The samples were tested at a 1:500 dilution for the three isotypes and 1/100 for the IgG subclasses. To quantify IgM and IgA, the samples and controls were pretreated with anti-human IgG (Gullsorb) at 1:10 dilution to avoid IgG interferences. Antibody levels, expressed as mean fluorescence intensity (MFI), were obtained using the Flexmap 3D^®^ reader (Luminex). The seropositivity cutoffs were calculated with the prepandemic plasma samples as 10 to the mean plus 3 SD of log_10_-transformed MFI values. Antibody data were log_10_-transformed to perform statistical analysis and visualization.

IgG avidity was also assessed and reported as the avidity index, which is determined as the percentage of IgG levels against NCT, NFL, RBD, S, and S2 antigens measured by incubating plasma samples with a chaotropic agent (urea 4 M, 30 min at room temperature) over the IgG levels measured in the same samples without a chaotropic agent.

### Statistical analysis

All analyses were performed in 1) all study participants with available samples at baseline, day 7, and day 60, defining the analysis population; and 2) all paired CCP units corresponding to the infused participants included in the analysis population. Descriptive data were summarized using medians [interquartile range (IQR)] or counts (%). The distributions of the assay data were skewed to the right, so they were transformed to a log_10_ scale for analysis.

The effect of treatment on antibody levels over time was assessed by evaluating differences in median antibody levels in participants between groups at baseline, day 7, and day 60 using the non-parametric Wilcoxon test. The same approach was also used to assess differences in the increase in antibodies according to participants’ serostatus at baseline and the neutralizing activity of infused CCP. Adjusted *p*-values were computed using the Benjamini and Hochberg method. Differences in the increase of median antibody levels at baseline, day 7, and day 60 were evaluated using the non-parametric paired signed rank test. The same was used to measure the effect of MB treatment in CCP units by comparing median antibody levels before and after MB treatment. Correlations were assessed between 1) the levels of each antibody isotype and subclass in infused CCP and those in recipients on day 7, 2) the increase in neutralizing antibody titers and antibody levels in recipients (between baseline and day 7), and 3) neutralizing antibody titers and the levels of each antibody isotype and subclass in MB-treated infused CCP. All correlations were examined using Spearman’s rank correlation coefficient. The multivariable relationship was explored between immune responses, measured as an increase from baseline to day 7 in antibodies, and clinical and virological outcomes, presented in a heatmap with hierarchical clustering in patients and antibodies. Clusters were constructed using the Ward minimum variance clustering method with Murtagh and Legendre criterion, implemented in the hclust R function under the “ward.D2” option. Differences in the ratios of antibody isotypes and subclasses in participants and infused CCP expressed in log_2_ were evaluated using non-parametric Wilcoxon tests. All analyses were performed using the R statistical package, version 4.3 or higher, at a significance level of 0.05.

We estimated that a sample size of 122 participants (61 per arm) would provide 80% power to detect a 50% reduction in the mean difference of neutralizing antibody levels between groups, assuming a two-sided type I error rate of 0.05. To account for a 10% loss to follow-up, we planned to enroll 135 participants.

## Results

### Characteristics of the study population

A total of 128/376 (34%) consecutively enrolled COnV-ert participants from the main study center with available baseline, day 7, and day 60 blood samples were included in this study: 68/188 (36%) participants who received MB-CCP infusion and 60/188 (32%) who received saline infusion as placebo (study flow chart, **Supplementary Figure S1**).

The baseline demographic and clinical characteristics were similar in the CCP and placebo groups (**Table 1**). The mean age was 58.7 [standard deviation (SD) 8.3] years, 55/128 (43%) were women, and 97/128 (75.8%) had at least one risk factor related to coexisting conditions. The median time from symptom onset to randomization and baseline sample collection was 4 (IQR 3 to 5) days. Baseline serum antibody status was established based on three different definitions and was considered negative in the following: 1) 111/128 (89%) participants with undetectable IgM and IgG based on results by chemiluminescence immunoassay; 2) 48/128 (38%) participants with undetectable IgG (total IgG and subclasses IgG1, IgG2, IgG3, and IgG4) and undetectable IgM and IgA, based on results by Luminex immunoassay and level thresholds ≤10 to the mean plus 3 SD of log_10_-transformed MFI values of negative prepandemic controls; and 3) 63/128 (50%) participants with undetectable neutralizing antibodies, defined as below the limit of detection plasma reciprocal dilution (ID_50_) <60, based on results by pseudovirus neutralization assay against the ancestral SARS-CoV-2 WH1. Hospitalization up to 28 days occurred in 14/128 (10.9%) participants, with 8/128 (6.2%) fulfilling the criteria of severe COVID-19 disease according to the WHO Clinical Progression Scale (scores 6 to 10). Viral load (VL) decrease >1 log_10_ from baseline to day 7 was observed in 101 (82.1%) participants.

**Table 1:** Demographic and clinical characteristics of study participants.

		Overall (n 128)	MB-CCP (n 68)	Placebo (n 60)
Demographic and clinical baseline characteristics				
Age in years—mean (SD)		58.7 (8.3)	58.3 (8.7)	59.1 (7.8)
Gender—n (%)	*Female*	55 (43.0)	26 (38.2)	29 (48.3)
	*Male*	73 (57.0)	42 (61.8)	31 (51.7)
BMI—median (IQR)		27.60 [24.50, 29.99]	27.73 [24.92, 29.99]	27.08 [24.12, 29.90]
At least one comorbidity—n (%)		97 (75.8)	48 (70.6)	49 (81.7)
Days to symptom onset—median (IQR)		4.0 [3.0, 5.0]	4.0 [3.0, 5.0]	4.0 [3.0, 5.3]
Days to positive test—median (IQR)		2.0 [2.0, 3.0]	3.0 [2.0, 3.0]	2.0 [2.0, 3.0]
Baseline antibody status A^*^—n (%)	*Negative*	111 (88.8)	58 (86.6)	53 (91.4)
	*Positive*	14 (11.2)	9 (13.4)	5 (8.6)
Baseline antibody status_B^†^—n (%)	*Negative*	48 (37.8)	27 (39.7)	21 (35.6)
	*Positive*	79 (62.2)	41 (60.3)	38 (64.4)
Baseline antibody status C^§^—n (%)	*negative*	63 (49.6)	27 (39.7)	36 (61.0)
	*Positive*	64 (50.4)	41 (60.3)	23 (39.0)
Clinical evolution—n (%)				
Hospitalization within 28 days		14 (10.9)	9 (13.2)	5 (8.3)
WHO Progression scale score >6 within 28 days		8 (6.2)	6 (8.8)	2 (3.3)
VL decrease of more than 1 log_10_ (baseline to D7)		101 (82.1)	54 (81.8)	47 (82.5)

MB-CCP, participants infused with methylene blue-treated COVID-19 convalescent plasma; SD, standard deviation; BMI, body mass index; IQR, interquartile range; MFI, mean fluorescence intensity.

^*^Baseline antibody status A: Based on results by chemiluminescence immunoassay and reported in the COnV-ert study (HUGTiP). Seronegative = IgG undetectable + IgM undetectable; Seropositive = rest of combinations (either IgG positive alone, IgM positive alone, or IgG + IgM positive).

^†^Baseline antibody status B: Based on results by Luminex dataset and defined by levels >10 to the mean plus 3 standard deviations (SD) of log_10_-transformed MFI values of prepandemic negative controls (ISGlobal). Seronegative = IgG undetectable + IgM undetectable + IgA undetectable; Seropositive = any positive response for any of the isotypes/antigen pairs.

^§^Baseline antibody status C: Based on neutralization assay. Negative = non-sero-neutralizers; Positive = sero-neutralizers.

### Anti-SARS-CoV-2 antibody levels in participants and increase according to MB-CCP or placebo administration

We measured anti-SARS-CoV-2 antibody levels at baseline (before infusion), day 7, and day 60 after infusion, including antibody isotypes IgG, IgM, and IgA and subclasses IgG1, IgG2, IgG3, and IgG4 against five different viral WH1 antigens NCT, NFL, RBD, S, and S2. We also assessed neutralizing antibody titers against the WH1 virus and IgG avidity.

**Figure 1:**
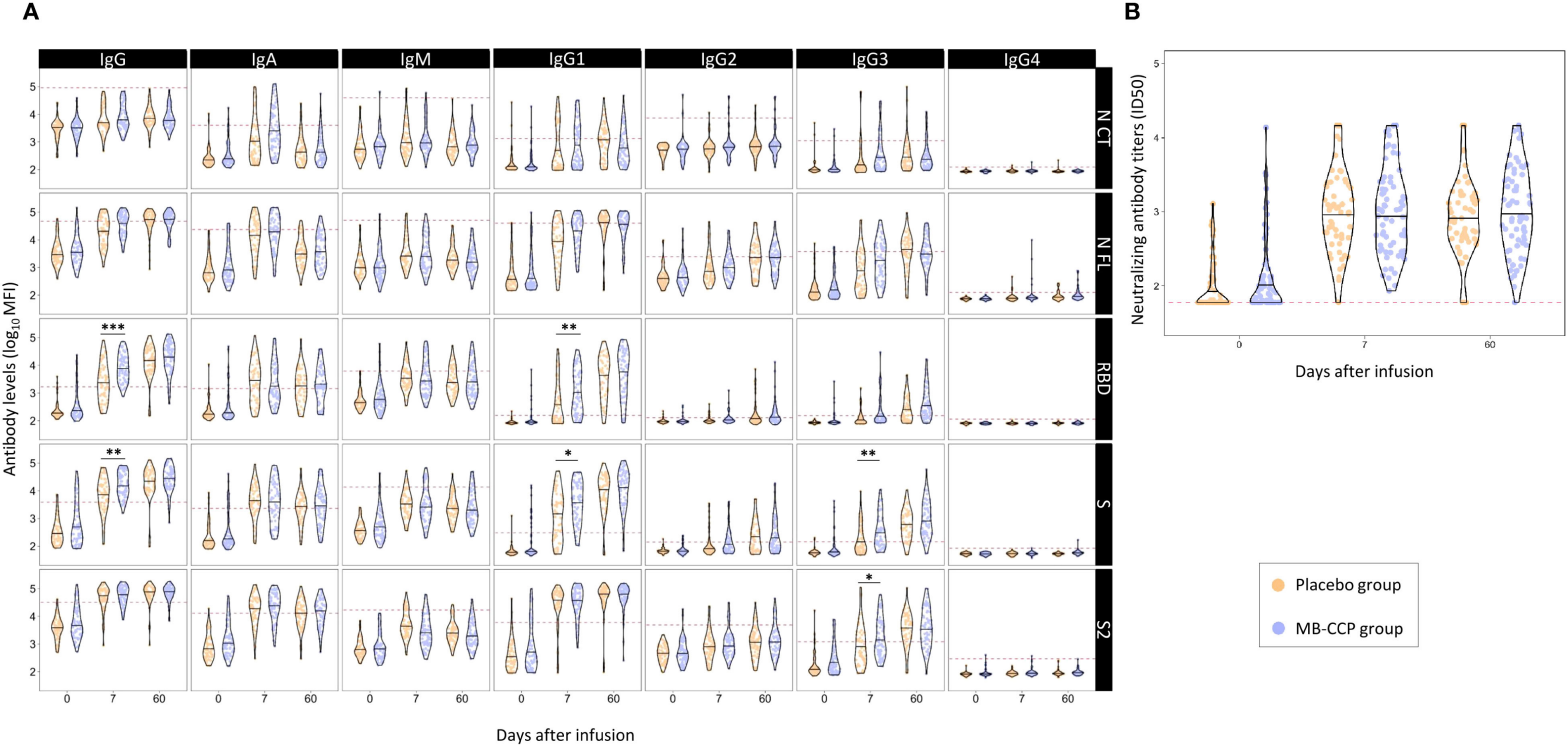
Distribution of anti-SARS-CoV-2 antibody levels in participants at baseline, day 7, and day 60 after infusion, according to trial group (MB-CCP and placebo). Legend: Violin plots of antibody levels in log_10_ in study participants in both groups [placebo group in orange (n = 60) and methylene blue-treated COVID-19 convalescent plasma group (MB-CCP) in blue (n = 68)] at baseline, day 7, and day 60 after infusion, with median (thick solid line). **(A)** Distribution of antibody isotypes (IgG, IgM, and IgA) and subclasses (IgG1, IgG2, IgG3, and IgG4) against five different viral target antigens [nucleocapsid C-terminal region (NCT), nucleocapsid full protein (NFL), receptor-binding domain (RBD), spike full protein (S), and S2 fragment (S2)] in median fluorescent intensity (log_10_ MFI) measured using Luminex. Red dotted line corresponds to the seropositivity cutoffs defined as 10 to the mean plus 3 standard deviations (SDs) of log_10_-transformed MFI values of 92 and 128 (IgA/IgM and IgG, respectively) prepandemic controls. **(B)** Distribution of neutralizing antibody titers (ID_50_) assessed by pseudovirus neutralization assay against Wuhan/WH1. Asterisks indicate statistically significant (*p < 0.05, **p < 0.01, and ***p < 0.001) differences in levels above the seropositivity cutoffs (based on *p*-values adjusted by time point). The full statistical description is shown in **Supplementary Table S1**. MB-CPP group, methylene blue-treated COVID-19 convalescent plasma group; ID_50_, 50% inhibitory dilution; MFI: mean fluorescence intensity. Geometric mean (geoM) values for significant level differences at day 7 (placebo group geoM vs. MB-CCP group geoM, geoM difference placebo − MB-CCP groups): RBD IgG 2,433.36 vs. 8,163.12, −5,729.76; RBD IgG1 395.05 vs. 1,073.85, −678.80; S IgG 6,535.20 vs. 16,619.74, −10,084.54; S IgG1 1,188.97 vs. 3,643.97, −2,355.00; S IgG3 178.12 vs. 354.55, −176.43; S2 IgG3 896.03 vs. 1,715.67, −819.63.

The distribution of anti-SARS-CoV-2 antibody levels in participants, including neutralizing antibody titers, at the three time points according to the trial group (MB-treated CCP and placebo) is shown in **Figure 1**. At baseline, there were no statistically significant differences in any antibody isotype or subclass between groups after adjusting *p*-values (**Figure 1**, **Supplementary Table S1**). However, unadjusted analyses revealed significantly higher, albeit very modest, neutralizing antibody titers in the MB-CCP group compared to the placebo group (difference in median ID_50_ titers 16.72; unadjusted *p*-value 0.041) (**Supplementary Table S1**).

Between baseline and day 7, an increase was observed for the levels of all antibody isotypes and subclasses, as well as neutralization, which was 5- to 11-fold for IgM, 27- to 50-fold for IgA, 5- to 50-fold for IgG, and 15-fold for neutralizing antibodies in the overall population (**Supplementary Table S2**). At day 7, the levels of IgG to RBD and S, IgG1 to RBD and S, and IgG3 to S and S2 were significantly higher in the MB-CCP group compared to placebo (**Figure 1**, **Supplementary Table S1**). The IgG avidity index was also significantly higher in the MB-CCP group compared to the placebo group for RBD, S, and S2 at day 7 (difference 0.16, *p* < 0.002; 0.15, *p* < 0.001; and 0.07, *p* = 0.045, respectively) (**Supplementary Table S1**) Interestingly, no significant differences were observed between treatment arms (MB-CCP and placebo) for neutralizing antibodies [median ID_50_ 1:1,017 (SD 2,029) vs. 1:989 (SD 1,825); *p* = 0.911] or for the levels of IgA, IgM, IgG2, or IgG4. Differences in the increase from baseline to day 7 were assessed according to 1) the participants’ serostatus at baseline based on results by Luminex [i.e., seronegative = IgG negative + IgG subclasses (IgG1, IgG2, IgG3, and IgG4) negative + IgM negative + IgA negative vs. seropositive = any positive response for any of the isotypes/antigen pairs] and 2) the neutralizing activity of infused MB-CCP. The increase between baseline and day 7 was significantly higher in participants who were seronegative at baseline for IgG to S and S2, IgG1 to S2, and IgA to S2 in the MB-CCP group alone, but not for neutralizing antibody titers (**Supplementary Figures S2, S3**). We did not observe significant differences in the levels of any antibody isotypes and subclasses or neutralizing antibodies, according to the neutralizing activity of MB-CCP infusion, which was stratified in ID_50_ <1,000, 1,000–5,000, and ≥5,000 (**Supplementary Figure S4**). These results indicate an increase between baseline and day 7, probably consistent with the development of the endogenous antibody response, with significant differences between treatment groups at day 7 in the levels of total IgG, IgG1, and IgG3, suggesting a contribution of infused antibodies from the MB-CCP unit.

The increase in levels between day 7 and day 60 was much smaller than that seen from baseline to day 7, mainly observed for total IgG and subclasses IgG1, IgG2, and IgG3, and more marked for the RBD and S antigens (**Figure 1**, **Supplementary Table S1**). By day 60, no significant differences in the levels of any of the measured antibody isotypes and subclasses, neutralizing antibodies, or IgG avidity index were shown between intervention groups. These results suggest that administering MB-CCP did not affect the mid-term host’s antibody response to SARS-CoV-2.

### Antibody responses to MB-CCP administration and correlations between antibody levels in donors and recipients

**Figure 2:**
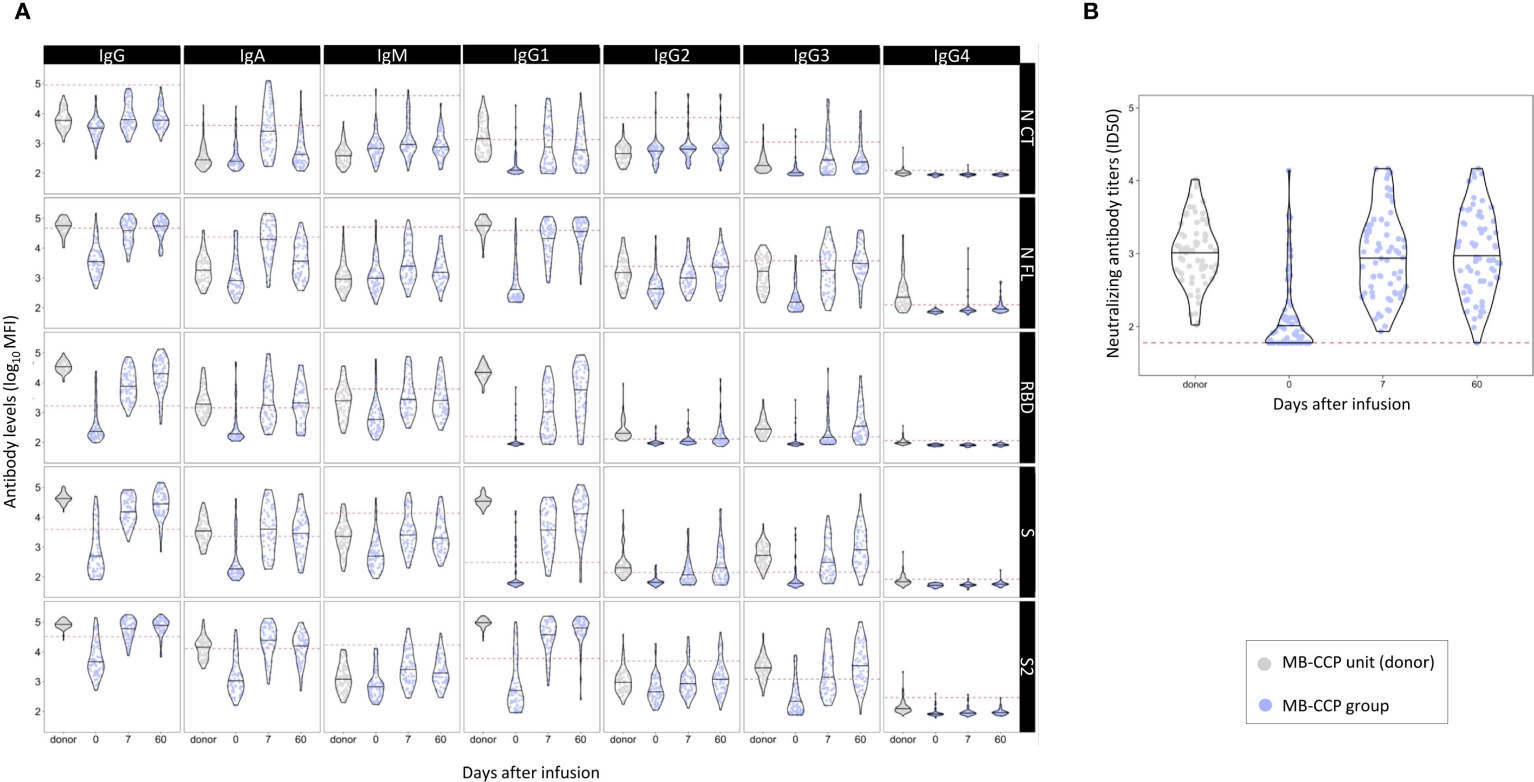
Distribution of anti-SARS-CoV-2 antibody levels in methylene blue-treated COVID-19 convalescent plasma (MB-CCP) units and in participants who received MB-CCP infusion (MB-CCP group) at baseline, day 7, and day 60 after infusion. Legend: Violin plots of antibody levels in log_10_ in methylene blue-treated COVID-19 convalescent plasma (MB-CCP) units and in study participants who received MB-CCP infusion at baseline, day 7, and day 60 after infusion (n = 68), with median (hick solid line). **(A)** Distribution of antibody isotypes (IgG, IgM, and IgA) and subclasses (IgG1, IgG2, IgG3, and IgG4) against five different viral target antigens [nucleocapsid C-terminal region (NCT), nucleocapsid full protein (NFL), receptor-binding domain (RBD), spike full protein (S), and S2 fragment (S2)] in median fluorescent intensity (log_10_ MFI) measured using Luminex. Red dotted lines correspond to the seropositivity cutoffs defined as 10 to the mean plus 3 standard deviations (SD) of log_10_-transformed MFI values of 92 and 128 (IgA/IgM and IgG, respectively) prepandemic controls. **(B)** Distribution of neutralizing antibody titers (ID_50_) assessed by pseudovirus neutralization assay against Wuhan/WH1. MB-CPP, methylene blue-treated COVID-19 convalescent plasma; ID_50_, 50% inhibitory dilution; MFI, mean fluorescence intensity.

The distribution of all anti-SARS-CoV-2 antibody levels analyzed in MB-treated CCP units and participants who received MB-CCP infusion at baseline, day 7, and day 60 after infusion is shown in **Figure 2**. Significantly higher levels of total IgG and all IgG subclasses, particularly IgG1, to NFL, RBD, S, and S2, were observed in MB-treated CCP units (donors) compared to the paired level in the recipients at day 7 (**Figure 2**, **Supplementary Table S3**). Similarly, the IgG avidity index was higher in the infused MB-treated CCP units than in their recipients at day 7. In contrast, participants exhibited higher levels of IgA and IgM at day 7 than those present in the infused units, with significant differences observed for antigens NCT, NFL, and S2. Neutralizing antibody titers were comparable between donors (infused MB-CCP units) and paired participants at day 7 (**Figure 2**, **Supplementary Table S3**).

**Figure 3:**
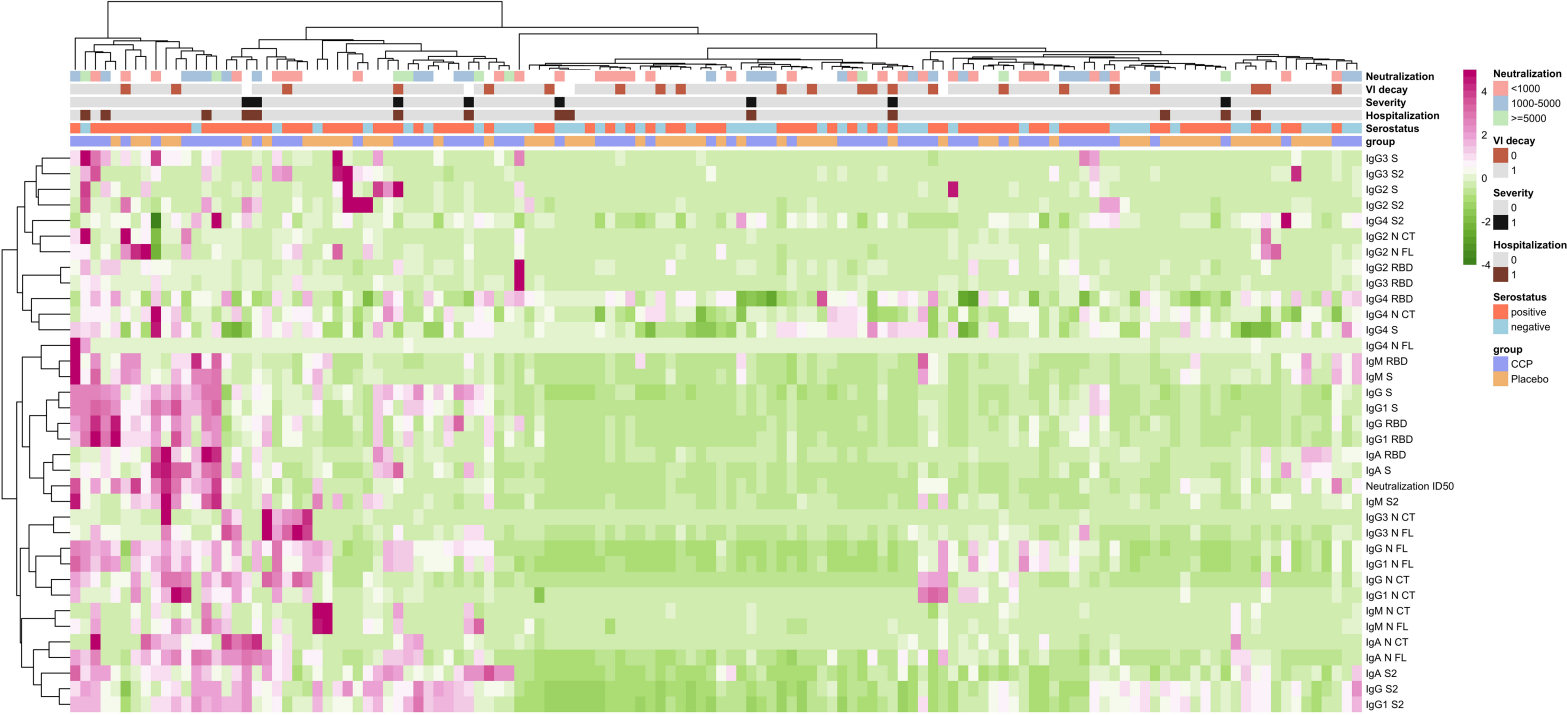
Heatmap of correlations between immune responses with receipt of MB-CCP and with clinical and virological outcomes. Legend: Heatmap with hierarchical clustering in patients and in antibodies, exploring multivariable relationship between immune responses, and clinical and virological outcomes. Immune responses were measured as increase from baseline to day 7 in all antibody levels, including isotypes IgG, IgM, and IgA and subclasses IgG1, IgG2, IgG3, and IgG4 measured using Luminex (in MFI), and neutralizing antibodies assessed by pseudovirus neutralization assay against Wuhan/WH1 (in ID_50_). Clinical outcomes included hospitalization within 28 days (0 = non-hospitalized; 1 = hospitalized) and severe COVID-19, defined as hospitalization with requirement of oxygen by non-invasive ventilation or high flow, intubation and mechanical ventilation, or death (WHO Clinical Progression Scale score 6 to 10) (0 = non-severe COVID-19; 1 = severe COVID-19). Virological outcome was defined as VL decay between baseline and day 7 (0 < 1 log_10_, 1 ≥ 1 log_10_). We also explored relationship with 1) neutralizing antibody titers contained in MB-CCP infusion (neutralization; stratified as neutralizing antibody titers ID_50_ <1,000, 1,000 to 5,000, and ≥5,000); 2) serostatus of participants at baseline based on results by Luminex (i.e., seronegative = IgG negative + IgG subclasses (IgG1, IgG2, IgG3, and IgG4) negative + IgM negative + IgA negative vs. seropositive = any positive response for any of the isotypes/antigen pairs); and 3) treatment group of participants (CCP = MB-CCP group; placebo = placebo group). Clusters were built using the Ward minimum variance clustering method with Murtagh and Legendre criterion, implemented in the hclust R function under the “ward.D2” option. MB-CCP, methylene blue-treated COVID-19 convalescent plasma.

We also assessed correlations between the levels of antibody isotypes and subclasses and neutralizing antibodies in MB-CCP units (donors), in participants who received MB-CCP infusion (MB-CCP group), and between both. In CCP units (donors), we observed a modest-to-strong significant correlation between neutralizing antibody titers and the levels of IgM to NCT, NFL, RBD, and S (Spearman’s ρ 0.54, 0.61, 0.57, and 0.66, respectively), IgA to S2 (ρ 0.48), and IgG and IgG1 to NCT (ρ 0.61 and ρ 0.63, respectively) (**Supplementary Figure S5**). However, no clear correlation was observed for neutralizing antibody titers and the levels of IgG to RBD and S, or EUROIMMUN ratio (ρ 0.17), which did show a good correlation with IgG to RBD and S (ρ 0.53 and 0.51, respectively). We observed a low correlation between MB-treated CCP units infused (donors) and recipients at day 7 between the levels of all antibody isotypes and subclasses and neutralization titers (**Supplementary Figure S6**). At day 7, MB-CCP recipients showed significant positive correlations (Spearman’s ρ > 0.5) between their neutralizing antibody titers with the levels of IgM to RBD, S, and S2; IgA to RBD and S; IgG to NCT, IgG1 NCT, RBD, and S; and IgG3 to NCT, NFL, RBD, and S2 (**Supplementary Figure S7**). These findings are further supported by the global analysis presented in **Figure 3**, which shows a close clustering of the levels of neutralizing antibodies and IgM to S2 and IgA to RBD and S in participants at day 7. All these results suggest a considerable dilution of antibodies contained in the MB-CCP product 7 days after infusion and a fast elicitation of endogenous IgM and IgA antibodies at day 7, which correlate with neutralizing antibodies from the newly generated responses.

### Correlations between immune responses with clinical and virological outcomes

We explored correlations between immune responses measured as an increase from baseline to day 7 in all isotypes and subclasses and neutralizing antibodies, with clinical and virological outcomes. We found no significant associations between immune responses and disease progression within 28 days, including hospitalization and development of severe COVID-19, defined as hospitalization with the requirement of oxygen by non-invasive ventilation of high flow, intubation and mechanical ventilation, or death (WHO Clinical Progression Scale score 6 to 10). Similarly, no correlations were observed between immune responses and virological outcome, measured as decline in viral load (log_10_) from baseline to day 7 (**Figure 3**). These data are in line with the COnV-ert results, showing no association of the administration of MB-CCP with clinical or virological outcomes measured.

### Effect of methylene blue treatment on the antibody levels in COVID-19 convalescent plasma units

**Figure 4:**
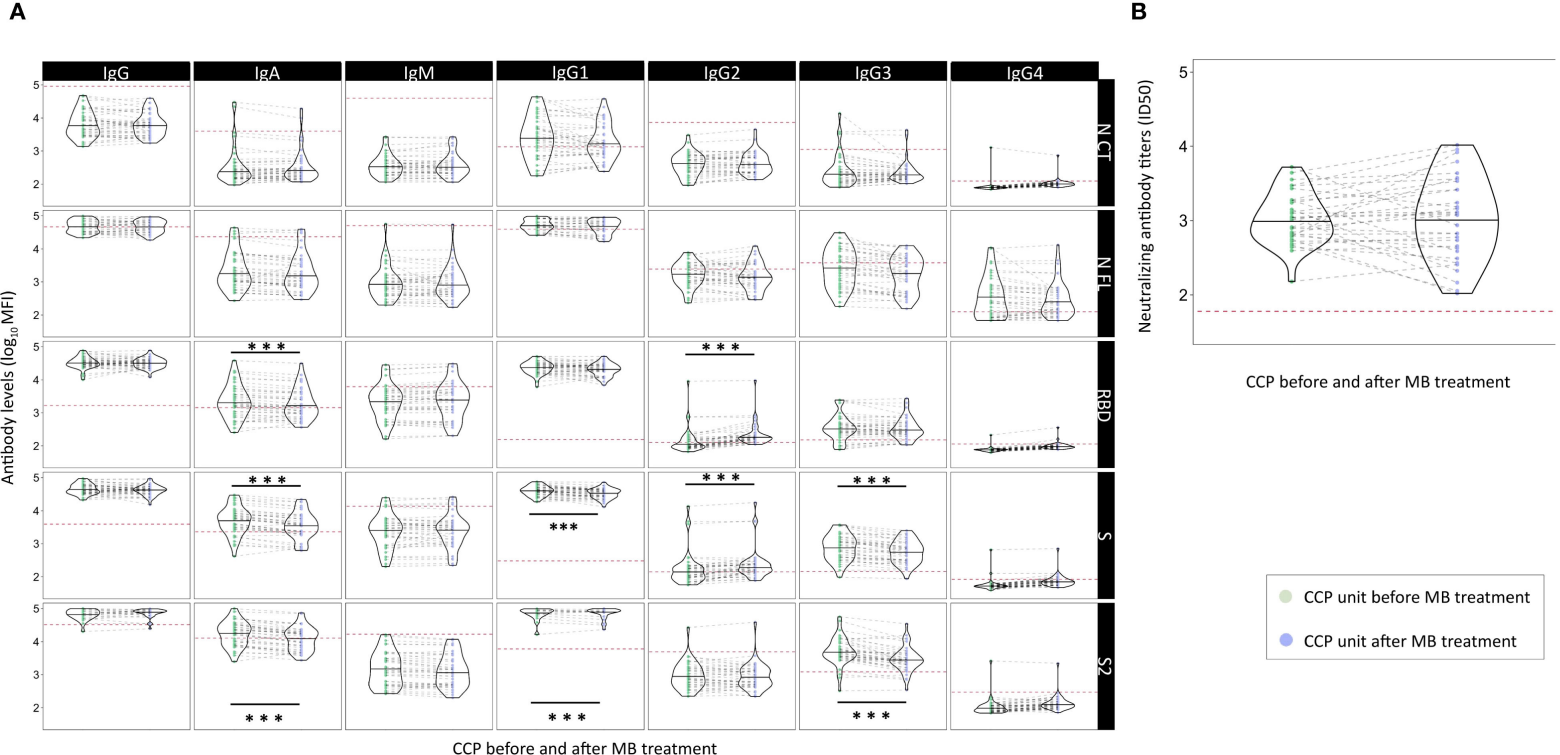
Effect of methylene blue treatment as a pathogen inactivation method for COVID-19 convalescent plasma on levels of SARS-CoV-2 neutralizing antibodies and antibody isotypes and subclasses. Legend: Violin plots of antibody levels in log_10_ in transfused convalescent plasma samples before and after methylene blue treatment (n = 40), with median (thick solid line). **(A)** Distribution of antibody isotypes (IgG, IgM, and IgA) and subclasses (IgG1, IgG2, IgG3, and IgG4) against five different viral target antigens [nucleocapsid C-terminal region (NCT), nucleocapsid full protein (NFL), receptor-binding domain (RBD), spike full protein (S), and S2 fragment (S2)] in median fluorescent intensity (log_10_ MFI) measured using Luminex. Red dotted lines correspond to the seropositivity cutoffs defined as 10 to the mean plus 3 standard deviations (SD) of log_10_-transformed MFI values 92 and 128 (IgA/IgM and IgG, respectively) prepandemic controls. **(B)** Distribution of neutralizing antibody titers (ID_50_) assessed by pseudovirus neutralization assay against Wuhan/WH1. Asterisks indicate statistically significant (*p < 0.05, **p < 0.01, and ***p < 0.001) differences in levels above the seropositivity cutoffs. The full statistical description is shown in **Supplementary Table S4**. Geometric mean values for significant level differences (post-MB geoM vs. pre-MB geoM, geoM difference post − pre-MB): RBD IgA 1,857.92 vs. 2,190.01, −332.09; RBD IgG2 221.70 vs. 140.71, 80.99; S IgA 3,503.13 vs. 4,888.37, −1,385.22; S IgG1 32,959.78 vs. 39,678.33, −6,718.55; S IgG2 235.37 vs. 172.36, 63.01; S IgG3 559.91 vs. 780.18, −220.27; S2 Ig1 12,786.28 vs. 17,985.83, −5,199.55; S2 IgG1 88,774.91 vs. 96,769.90, −7,994.99; S2 IgG3 2,938.25 vs. 5,099.03, −2,160.79.

Only pre-selected CCP units with high anti-SARS-CoV-2 IgG titers (EUROIMMUN ratio ≥6) were infused in the COnV-ert study, after treatment with MB as a pathogen inactivation method. To assess the effect of MB treatment, we measured anti-SARS-CoV-2 antibody levels, including antibody isotypes and subclasses against the five SARS-CoV-2 antigens, as well as neutralizing antibodies, in 40 CCP paired units before and after treatment with MB. After MB treatment, we observed significantly lower levels of IgA to RBD [median MFI 1,551 (IQR 3,064.50) vs. 1,890.55 (IQR 3,086.80) before MB treatment, *p* < 0.001], S [median MFI 3,726 [IQR 4,077] vs. 5,775 [IQR 5,612.39] pre-MB, *p* < 0.001], and S2 [median MFI 14,868.50 (IQR 14,575.75) vs. 19,147.82 (IQR 24,518.78) pre-MB, *p* < 0.001]; IgG1 to S [median MFI 35,485.50 (IQR 151,14.75) vs. 41,685.51 (IQR 18,510.83) pre-MB, *p* = 0.001] and S2 [median MFI 93,975 (IQR 42,124) vs. 109,843.97 (IQR 38,433.06) pre-MB, *p* < 0.001]; and IgG3 to S [median MFI 560 (IQR 707.75) vs. 870.16 (IQR 1,100.07) pre-MB, *p* < 0.001] and S2 [median MFI 2,858 (IQR 2,454.50) vs. 4,587 (IQR 5,851.64) pre-MB, *p* < 0.001] (**Figure 4**, **Supplementary Table S4**). We also observed a significant but much smaller increase in IgG2 to S [median MFI 187.50 (IQR 117.50) vs. 130.70 (IQR 112.24) pre-MB, *p* < 0.001] and RBD [median MFI 179 (IQR 86) vs. 103.34 (IQR 72.30) pre-MB, *p* < 0.001], and similar levels of IgM, IgG, IgG2, IgG4, and neutralizing antibodies after MB treatment [median neutralizing antibody titers ID_50_ 1:1,192 (SD 1:2,378) vs. 1:1,053 (SD 1:1,226) before MB treatment; *p* = 0.077] (**Figure 4**, **Supplementary Table S4**). These results suggest a potential adverse effect of MB on the antibodies contained in the CCP units, with a significant and consistent decrease in IgA, IgG1, and IgG3 to S and S2.

### Ratios of antibody isotypes and subclasses in participants and MB-CCP infusion

**Figure 5:**
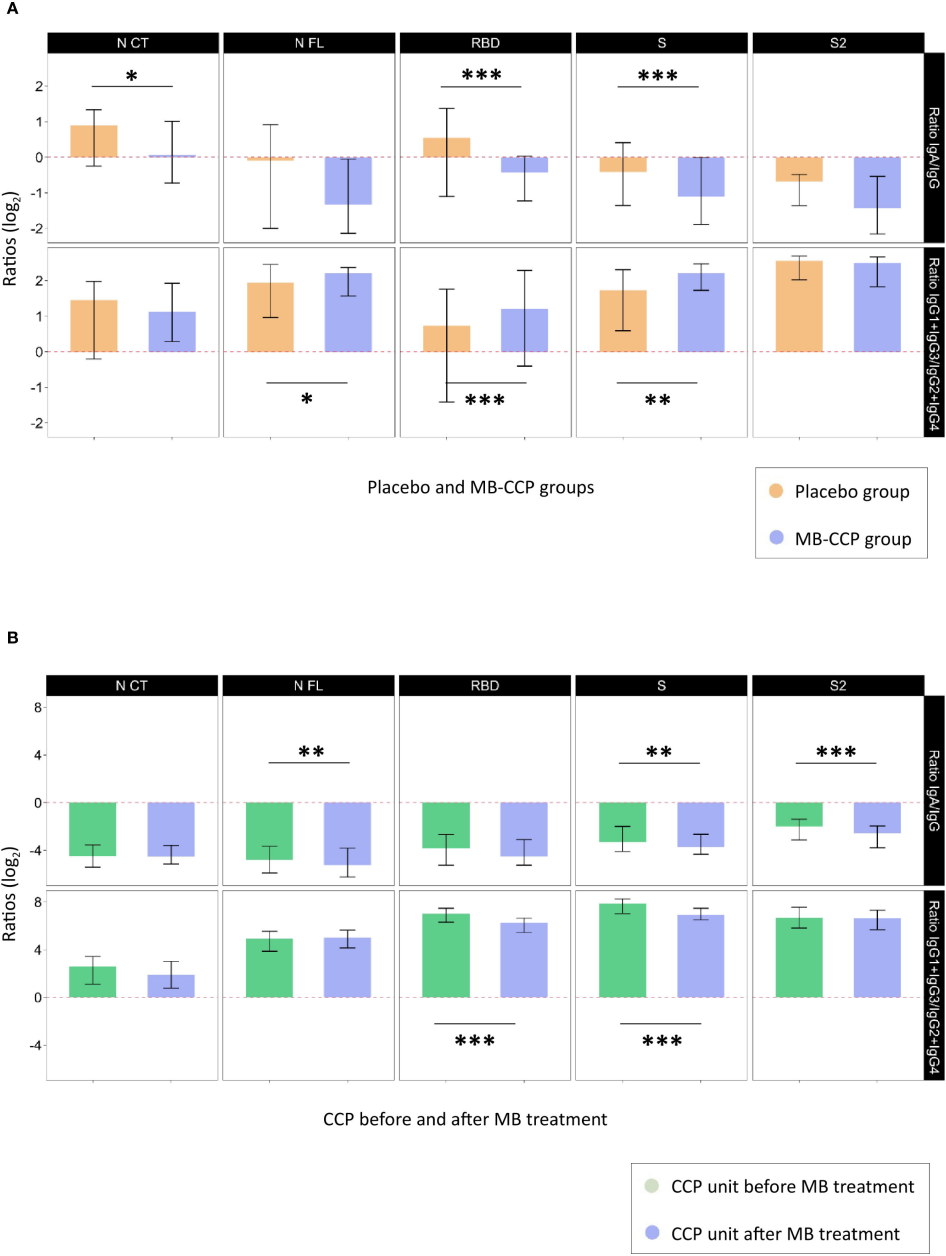
Ratios of IgA/IgG isotypes and cytophilic over non-cytophilic IgG subclasses. Legend: Ratios of IgA/IgG isotypes and cytophilic over non-cytophilic IgG subclasses (IgG1 + IgG3/IgG2 + IgG4) for each of the antigens (NCT, NFL, RBD, S, and S2) that could reflect differential non-neutralizing Fc-related functional characteristics, expressed in log2. **(A)** Study participants at day 7 after infusion according to treatment group [placebo (n = 60) and MB-CCP groups (n = 68)]. **(B)** COVID-19 convalescent plasma units before and after treatment with methylene blue (n = 40). Asterisks indicate statistically significant (*p < 0.05, **p < 0.01, and ***p < 0.001) differences in ratios calculated with antibody levels above the seropositivity cutoffs. The full statistical description is shown in **Supplementary Table S5**. NCT, nucleocapsid C-terminal region; NFL, nucleocapsid full protein; RBD, receptor-binding domain; S, spike full protein; S2, S2 fragment; MB-CCP, methylene blue-treated COVID-19 convalescent plasma.

We assessed ratios of IgA/IgG isotypes and cytophilic over non-cytophilic IgG subclasses (IgG1 + IgG3/IgG2 + IgG4) for each of the antigens (NCT, NFL, RBD, S, and S2) in 1) study participants according to treatment group at day 7 after infusion and 2) CCP units before and after MB treatment. Regarding participants at day 7, the MB-CCP group showed a smaller ratio of IgA/IgG that was statistically significant for NCT, RBD, and S; and a larger ratio of cytophilic over non-cytophilic IgG subclasses, significant for NFL, RBD, and S compared to the placebo group (**Figure 5A**, **Supplementary Table S5**). These results are in line with the higher levels of IgG, IgG1, and IgG3 observed for participants receiving MB-CCP at day 7 in **Figure 1**, suggesting an impact of infusions on the IgG ratios. Following MB treatment, CCP units showed lower IgA/IgG ratios and cytophilic/non-cytophilic IgG subclass ratios compared to pre-MB treatment, with significant reductions for NFL, S, and S2 (IgA/IgG) and for RBD and S (IgG subclass ratios) (**Figure 5B**, **Supplementary Table S5**). These results suggest a potential decrease in IgA and cytophilic subclasses (IgG1 + IgG3) due to MB treatment, in line with data observed in **Figure 4**.

## Discussion

In this secondary analysis of our double-blind, placebo-controlled RCT including 128 (34%) of the outpatients with COVID-19 enrolled in the COnV-ert study, we observed that intravenous infusion of 1 unit of MB-treated high titer CCP significantly increased anti-SARS-CoV-2 IgG and IgG1 (to RBD and S), IgG3 (to S and S2), and IgG avidity in recipients at day 7 after infusion without an increase in IgA, IgM, IgG2, IgG4, or neutralizing antibodies. Moreover, this increase was significantly higher in participants who were seronegative at baseline for total IgG (to S and S2) and IgG1 (to S2) in the MB-CCP group, but not for anti-RBD IgG1 and IgG3. Importantly, levels observed in the recipients at day 7 compared to the paired MB-CCP units infused were lower for IgG, all IgG subclasses, and IgG avidity index; similar for neutralizing antibodies; and higher for IgA and IgM. We hypothesize that the observed post-infusion increase in IgG subclasses and the avidity index is a direct consequence of MB-CCP infusion. The lack of parallel changes in neutralization titers may be explained by the rapid elicitation of neutralizing antibodies in SARS-CoV-2-infected individuals (26), which do not require high-affinity maturation. Our results further suggest a considerable dilution of antibodies in recipients and a fast development of endogenous IgA and IgM by day 7, which correlated with the levels of neutralizing antibodies from the newly generated responses. Our study did not find correlations between immune responses of participants (MB-CCP and placebo group) and clinical or virological outcomes, in line with the results from the COnV-ert trial showing no differences between arms in hospitalization within 28 days or viral load reduction within 7 days. Furthermore, our findings indicate that MB-CCP administration did not positively or negatively impact the host antibody response to SARS-CoV-2, as levels at day 60 did not differ between the MB-CCP and placebo groups.

Five RCTs have evaluated the efficacy of CCP in unvaccinated outpatients with COVID-19, yielding mixed results. Two trials [CCP-Argentina (12) and CSSC-004 (14)] demonstrated a significant reduction in disease progression or hospitalization risk, two others [COnV-ert (10) and C3PO (11)] found no clinical efficacy, and a fifth trial [COV-Early (13)] suggested potential efficacy that did not reach statistical significance. Understanding the factors underlying these discrepancies is essential for optimizing the use of polyclonal antibody therapies for COVID-19 and other emerging viral infections. One key difference across trials was the variability in the SARS-CoV-2 antibody levels and functional properties within infused CCP. While all trials administered high-titer CCP, in accordance with FDA guidelines, differences in antibody functionality testing methodologies (including binding and virus-neutralizing antibody assays) complicate the comparability of CCP efficacy across studies. This variability further challenges efforts to define the role of neutralizing capacity in CCP clinical efficacy.

Antibody responses following CCP transfusion have been characterized in secondary analysis of some of the aforementioned trials on outpatients. A sub-analysis of the C3PO trial observed a two-fold increase in the levels of S-specific antibodies and neutralizing antibody activity 1 h after infusion in CCP recipients compared to placebo, which was significant only for participants who were seronegative at baseline (27). Notably, considerable dilution of the CCP product after administration was reported, with comparable antibody levels between groups at days 15 and 30, which were significantly higher than those immediately after infusion. Similarly, a prespecified analysis from the CSSC-004 trial, including only unvaccinated seronegative outpatients, demonstrated significantly higher levels of anti-S-RBD IgG 30 min after infusion in CCP recipients compared to controls (28). They also reported a 21.3-fold dilution of antibodies contained in the CCP after administration and comparable IgG levels between groups at days 14, 28, and 90 post-infusion. These findings suggest that CCP transfusion has a modest impact on circulating antibody levels compared to those generated by the host immune response during the following weeks and are consistent with our study results.

An important difference of the COnV-ert study was the use of MB as a pathogen inactivation method applied to infused CCP. Our findings suggest that MB treatment was associated with a significant consistent decrease in the levels of cytophilic subclasses IgG1 and IgG3 (to S and S2) and IgA (to RBD, S, and S2) in the CCP units, without a reduction in neutralizing antibody titers or the rest of the antibodies analyzed. Additionally, we observed a smaller increase in IgG2 levels, consistent with RBD and S, which could be a collateral effect of the decrease in IgA, IgG1, and IgG3 antibodies due to reduced competition for antigen-binding sites. Pathogen reduction methods, including amotosalen, riboflavin, and MB, have been suggested to negatively impact the integrity of antibodies present in CP (16). These technologies generate reactive oxygen species, which can oxidize proteins and glycosylated structures, potentially altering antibody structure and function. Such oxidative effects may damage critical domains or glycan residues involved in Fc-mediated immune responses (29, 30). However, the overall impact of these inactivation technologies on CCP remains uncertain, with studies reporting conflicting findings on antibody impairment and limited evidence regarding their effects on Fc effector functions. A study of 35 plasma samples showed no reduction in anti-SARS-CoV-2 RBD or S1 IgG and neutralizing antibodies following MB treatment (19). Another study comparing different pathogen reduction methods in CCP reported a small significant reduction in neutralizing antibodies and anti-RBD IgG, but not anti-S + N IgG or IgM, with MB, although the effect was less pronounced than with amotosalen or riboflavin (18). Psoralen/UV light and amotosalen pathogen inactivation methods did not significantly affect SARS-CoV-2 IgG antibody levels, neutralizing capacity, or antigen-binding properties in CCP (31, 32). Additionally, a smaller study with 10 paired plasma samples showed no significant changes in antigen-binding capacity for anti-EBV and anti-tetanus toxin IgG, nor in IgG Fc receptor binding, before and after MB treatment (33). A recent *ad hoc* sub-study of the ConPlas-19 trial, which showed a benefit of CCP in preventing respiratory deterioration or death when administered early in hospitalized COVID-19 patients, observed no clinical differences between participants who received CCP treated with MB and CCP treated with other inactivation methods (riboflavin or amotosalen) (34).

Our study indicates a potential negative effect of MB on IgG1 and IgG3 cytophilic subclasses, which have been shown to correlate with Fc-mediated effector functions (35). It is possible that the observed increase in cytophilic subclasses among recipients would have been significantly higher without MB treatment, which may have contributed to the negative results of the COnV-ert trial. While neutralizing antibody titers are considered the primary mediators of CCP’s antiviral effects (36), non-neutralizing antibodies have also been shown to contribute through Fc-dependent functions, such as Antibody-Dependent Cell-mediated Cytotoxicity (ADCC), Complement-Dependent Cytotoxicity (CDC), and phagocytosis (35). Animal model studies have shown that monoclonal antibodies (mAbs) against SARS-CoV-2, in addition to direct neutralization, require intact Fc effector functions for optimal *in vivo* efficacy (37–40). While in pre-exposure settings, mAbs may primarily rely on neutralization to prevent initial viral infection and limit dissemination, in the therapeutic settings, additional mechanisms mediated by Fc effector functions may become essential for viral clearance, inflammation control, and tissue repair (37). Evidence on effector mechanisms contributing to the antiviral activity of polyclonal antibodies raised in the context of vaccination has also been described in animal models (41, 42) and humans (43, 44). Increase in IgG2 following booster vaccination has been associated with poorer Fc effector functionality and decreased neutralization and protective immunity (45). Additionally, the CONCOR-1 trial suggested that the antibody profile significantly modified the effect of CCP on clinical outcomes (46). In line with our findings, this supports the hypothesis that CCP delivered in lower virus-specific antibody doses (1–2 and 54 mg in CSSC-004 (14, 28) and EAP BARDA (47) trials, respectively) may be more reliant on non-neutralizing effector functions for clinical efficacy, in contrast to monoclonal antibodies, which are typically given at 10–100 times higher neutralizing doses.

Interestingly, neutralizing antibody titers in MB-treated CCP units infused in our trial correlated well with IgM and IgA, but not with IgG (to RBD, S, and S2), or the EUROIMMUN ratio (which correlated with each other). These results contrast with earlier reports showing a strong correlation between anti-S IgG antibodies and neutralization (48, 49), which informed the donor emergency use authorization criteria for high-titer CCP (from unvaccinated donors) and the selection of CCP units in the COnV-ert trial. This evidence has been widely replicated, including in the secondary analysis of the CSSC-004 trial (28). However, in line with our findings, other studies have identified IgM and IgG3 as the primary isotypes and subclasses driving virus neutralization in CCP from unvaccinated donors (50, 51). In contrast, CCP from vaccinated donors exhibits significantly higher neutralizing and Fc-mediated activities, with neutralization less dependent on IgM and more reliant on IgG, likely due to the maturation of adaptive immune responses (52).

Our study has several limitations. First, the assessment of immune responses in MB-CCP recipients within the first 24 to 72 h after infusion was not possible, given the timing of sample collection. It is likely that by day 7 post-transfusion, the measured antibody levels likely represent a mixture of passive antibodies and recipient-generated antibodies, which could have confounded our results. Importantly, in the absence of clinical efficacy, the lack of observed effects (no change in antibody levels) is difficult to interpret. Second, the current study did not include all randomized participants and infused CCP units from the COnV-ert trial, limiting the generalizability of the results to the entire cohort. Nevertheless, we analyzed 128 of the first 135 consecutive participants with available samples recruited at the main study center, which accounted for over 73% of total enrollment, making this subgroup likely representative of the overall trial population. Third, the study included only unvaccinated individuals enrolled prior to the emergence of the Omicron variants, most of whom were immunocompetent. This limits the generalizability of our findings to immunocompromised individuals with prior infections and full vaccination, who currently benefit the most from treatment with polyclonal passive immunotherapies (6, 53, 54). Fourth, while we assessed antigen binding, avidity, and neutralizing function, we did not evaluate the integrity of the antibodies’ Fc region, which is large and potentially vulnerable to direct oxidative damage (17, 20), nor did we directly assess Fc-dependent functions through functional antibody response assays. However, there is robust evidence suggesting that Fc-mediated effector functions most strongly correlate with FcγR-binding antibodies and with IgG1 and IgG3 antibodies, which strongly ligate FcγR (35, 45). An additional limitation is that we did not assess the lymphocyte responses following CCP infusion. Fifth, all CCP units infused were collected from convalescent donors prior to the vaccination campaign and the emergence of the Omicron variants. As such, our findings regarding both immune responses in recipients and the effect of MB treatment in CCP units may not be applicable to currently available fully vaccinated and post-Omicron plasma (Vax-CCP), which has neutralizing antibody titers 10 times higher than those of pre-Omicron CCP (55, 56). Sixth, as all our study participants received MB-treated CCP, we could not compare immune responses with non-MB-treated CCP infusion, limiting the strength of our conclusions.

In conclusion, we observed a modest impact of a single intravenous infusion of MB-treated high-titer CCP (from unvaccinated donors) on circulating antibody levels compared to those generated by the host immune response by day 7. While IgG, IgG1, and IgG3 at day 7 were significantly higher in CCP recipients than in controls, IgA, IgM, and neutralizing antibody titers remained comparable, suggesting a peak in the endogenous humoral response. Importantly, our study points to a limited but negative effect of MB on IgG1 and IgG3, which are essential for effector functions beyond neutralization, without compromising neutralizing antibody titers. Future studies should focus on the direct evaluation of the impact of MB on Fc-dependent functions, especially in vax-CCP, which contains substantially higher levels of antibodies with neutralizing and other effector functions. Additionally, further research is needed to determine whether these effects influence the therapeutic efficacy of vax-CCP, particularly in immunosuppressed individuals who currently benefit most from this therapy. These findings could guide the optimization of pathogen inactivation protocols and inform the selection of the preferred technologies to preserve both quality and effectiveness of CCP. Addressing these questions is also essential for preparing for future viral epidemics, where convalescent plasma may serve as a first-line treatment before vaccines and specific therapeutics become available.

## Data Availability

The raw data supporting the conclusions of this article will be made available by the authors, without undue reservation.
