# Supplementary material for: Patient and donor antibody profiles in early COVID-19 convalescent plasma therapy in the COnV-ert trial
Source: Front Immunol. 2025 Sep 25;16:1647488. doi: 10.3389/fimmu.2025.1647488 (PMC12507808; doi:10.3389/fimmu.2025.1647488)
Supplement: Supplementary file 1 [file Table1.pdf]

Supplementary appendix to Patient and donor antibody profiles in early COVID-19 convalescent plasma therapy in the COnV-ert trial

Table of contents

COnV-ert Group of Authors 2

Figure S1. Study flowchart. 3

Table S1: Distribution of antibody levels in participants at baseline, day 7, and day 60 after infusion, according to trial group (MB-CCP and placebo) 4

Table S2: Increase in levels of antibodies between baseline and day 7 and day 60 in the overall population 8

Figure S2: Increase in antibody levels from baseline to day 7 in overall study participants (MB-CCP and placebo groups) stratified by serostatus of participants at baseline 9

Figure S3: Increase in antibody levels from baseline to day 7 in participants from MB-CCP group stratified by serostatus of participants at baseline 11

Figure S4: Increase in antibody levels from baseline to day 7 in participants from MB-CCP group stratified by neutralizing activity of convalescent plasma infused 13

Table S3: Distribution of anti-SARS-CoV-2 antibody levels in methylene blue-treated COVID-19 convalescent plasma (MB-CCP) units and in participants that received MB-CCP infusion (MB-CCP group) at day 7 after infusion 15

Figure S5: Correlation between neutralizing antibody titers and antibody levels in MB-treated CCP (donors)..16

Figure S6. Correlation between antibody levels in MB-CCP (from donors) and in participants treated with MB-CCP at day 7. 18

Figure S7: Correlation between neutralizing antibody titers and antibody levels in participants treated with MB-CCP from baseline to day 7..21

Table S4: Effect of Methylene Blue treatment for COVID-19 convalescent plasma on levels of SARS-CoV-2 neutralizing antibodies and antibody isotypes and subclasses. 23

Table S5: Ratios of IgA/IgG and cytophilic over non-cytophilic IgG subclasses (IgG1+IgG3 / IgG2 + IgG4) for each of the antigens (N_CT, N_FL, RBD, S, S2) in study participants at day 7 according to treatment group (placebo and MB-CCP groups) and in CCP units before and after MB-treatment 24

COnV-ert Group of Authors

Fight Infectious Diseases Foundation, Badalona, Spain

Gèlia Costes, MD; Mar Capdevila-Jáuregui, MD; Pamela Torrano-Soler, RN; Alba San José, RN; Bonaventura Clotet, PhD, Prof.

Hospital Universitari Germans Trias i Pujol, Badalona, Spain

Glòria Bonet Papell, MD

Emergency Department, Bellvitge University Hospital, Hospital de Llobregat, Spain

Pierre Malchair, MD; Aurema Otero, MD; Jose Carlos Ruibal Suarez, MD; Alvaro Zarauza Pellejero, MD; Ferran Llopis Roca, PhD; Orlando Rodriguez Cortez, MD; Vanesa Garcia Garcia, RN

Unitat de Suport a la Recerca de la Catalunya Central, Fundació Institut Universitari per a la recerca a l'Atenció Primària de Salut Jordi Gol i Gurina, Sant Fruitós de Bages, Spain

Anna Ruiz-Comellas, PhD; Anna Ramírez-Morros, MPH; Josep Vidal-Alaball, PhD

Salut Catalunya Central, Hospital de Berga, Berga, Spain

Joana Rodríguez Codina, RN; Rosa Amado Simon, MD

IrsiCaixa

Silvia Marfil; Benjamin Trinité, PhD

Figure S1. Study flowchart.

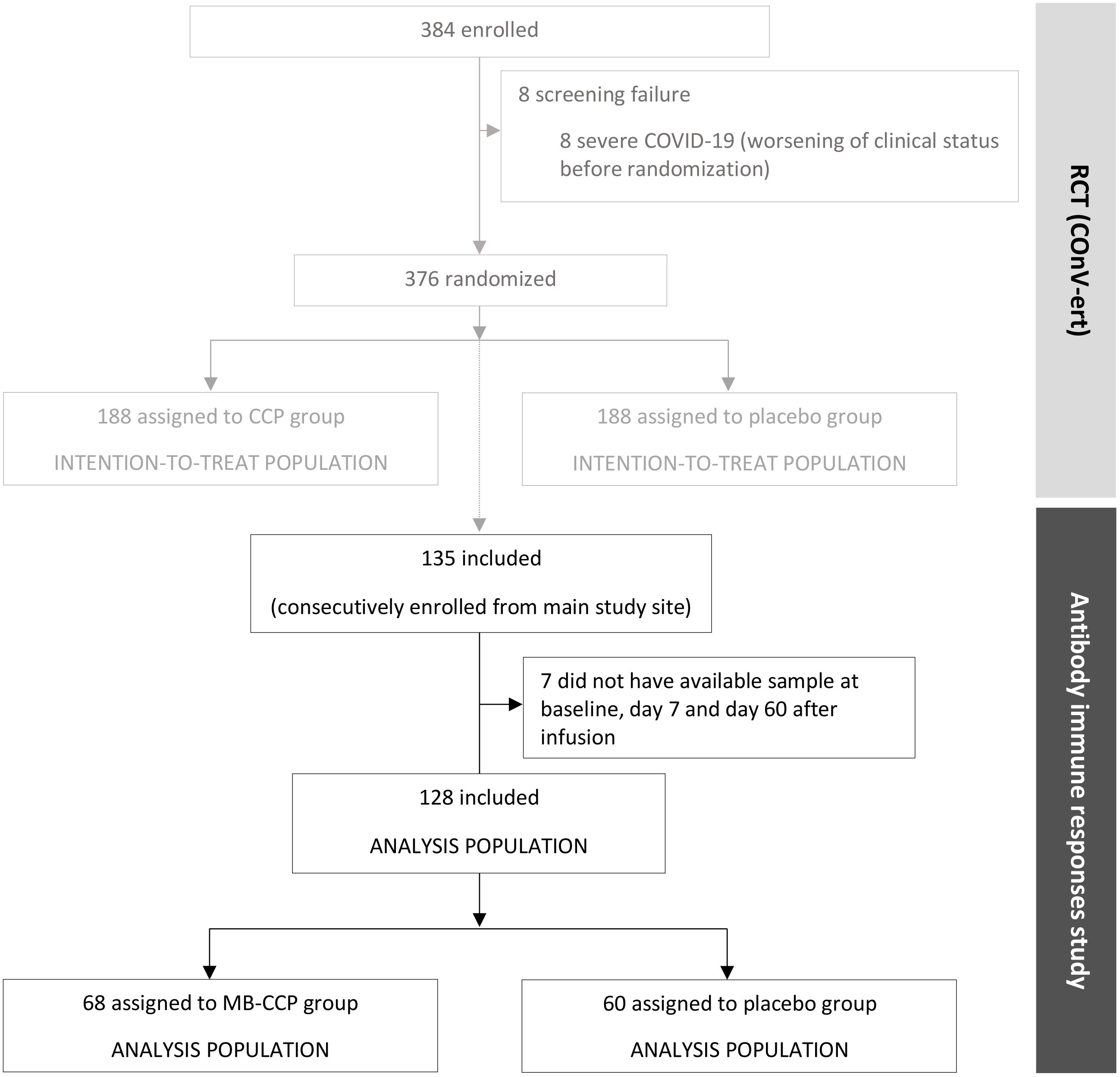

Table S1: Distribution of antibody levels in participants at baseline, day 7, and day 60 after infusion, according to trial group (MB-CCP and placebo)

Visit	Ag	Ab	Overall (n=128)	MB-CCP group (n=68)	Placebo group (n=60)	Placebo – MB-CCP	*p*-value	Adj *p*-value
Baseline	WH1	ID50	63.89 (73.71)	76.72 (75.06)	60 (58.35)	-16.722	**0.041**	0.243
	N CT	IgG	3511 (2684.25)	3452.5 (2805.88)	3528 (2435.5)	75.5	0.862	0.888
		IgA	214 (188)	216.25 (277.5)	214 (123)	-2.25	0.254	0.576
		IgM	598 (871.75)	685.75 (688.38)	493 (924.75)	-192.75	0.342	0.648
		IgG1	119 (59)	117.5 (56.5)	120 (58.5)	2.5	0.669	0.888
		IgG2	567 (421.75)	577.5 (438.38)	566 (414.5)	-11.5	0.869	0.888
		IgG3	97 (21)	100 (35.88)	97 (19)	-3	0.555	0.867
		IgG4	86.5 (7.5)	87.25 (9)	86 (6.75)	-1.25	0.571	0.867
	N FL	IgG	3254 (4958.5)	3425.5 (6009.38)	2748.5 (4175)	-677	0.520	0.867
		IgA	654 (1536)	692 (1841.38)	582 (887.5)	-110	0.341	0.648
		IgM	1002 (1245)	1003.5 (1453.5)	964.5 (1191)	-39	0.753	0.888
		IgG1	286 (751)	279.5 (845.38)	313 (572.75)	33.5	0.695	0.888
		IgG2	423 (399.5)	430.5 (482.88)	404 (324)	-26.5	0.629	0.888
		IgG3	121.5 (137.25)	125.25 (171.75)	118 (117.75)	-7.25	0.256	0.576
		IgG4	74 (9)	74 (10.13)	74 (8.25)	0	0.578	0.867
	RBD	IgG	187 (175)	207 (309.88)	181 (80.75)	-26	0.185	0.524
		IgA	170 (136.25)	170.5 (178.75)	168.5 (82)	-2	0.281	0.595
		IgM	489 (689.25)	568.5 (963.63)	444 (361)	-124.5	0.255	0.576
		IgG1	87.5 (11.25)	89 (11.5)	84.5 (9.5)	-4.5	0.003	0.119
		IgG2	94 (11.5)	94 (11.25)	93 (11.5)	-1	0.888	0.888
		IgG3	87 (9.75)	88 (11)	85.5 (8)	-2.5	0.189	0.524
		IgG4	81 (7.25)	80.5 (7.13)	81 (7.25)	0.5	0.799	0.888
	S	IgG	289 (767)	426 (1964.63)	284 (390.5)	-142	0.116	0.523
		IgA	152 (219)	167.5 (228.13)	143 (208.75)	-24.5	0.116	0.523
		IgM	397 (512.75)	475.75 (903)	350 (408.25)	-125.75	0.165	0.524
		IgG1	61 (16.5)	63 (18.38)	59 (14)	-4	0.021	0.243
		IgG2	66 (13.25)	66 (14.75)	66 (11)	0	0.885	0.888
		IgG3	58.5 (15.75)	59.5 (22.25)	57.5 (10.75)	-2	0.036	0.243
		IgG4	52 (9)	52 (9.63)	53 (8)	1	0.667	0.888
	S2	IgG	4066 (6039.75)	4533.25 (12715.38)	3864.5 (4000.25)	-668.75	0.167	0.524
		IgA	779 (1450.75)	1005.25 (1891.5)	623.5 (1132.75)	-381.75	0.012	0.221
		IgM	635.5 (728.5)	686 (733)	615 (683.5)	-71	0.879	0.888
		IgG1	341 (949)	365 (1436.25)	267.5 (596)	-97.5	0.159	0.524
		IgG2	447.5 (591.5)	409 (607)	448.5 (573)	39.5	0.873	0.888
		IgG3	130.5 (202.5)	163.5 (420)	117 (78)	-46.5	0.040	0.243
		IgG4	81.5 (9.5)	82.75 (8.75)	81 (10.75)	-1.75	0.387	0.696
	N CT	IgG	0.29 (0.25)	0.19 (0.05)	0.58 (0.24)	0.39	0.121	0.607

	N FL	AI	0.33 (0.19)	0.31 (0.23)	0.35 (0.18)	0.04	0.637	0.761
	RBD		0.12 (0.14)	0.17 (0.14)	0.08 (0.03)	-0.09	0.301	0.685
	S		0.10 (0.2)	0.10 (0.15)	0.11 (0.20)	0.01	0.761	0.761
	S2		0.54 (0.28)	0.54 (0.31)	0.53 (0.16)	-0.01	0.411	0.685
Day 7	ID50	ID50	990.00 (1926.28)	1017.1 (2029.35)	988.85 (1825.11)	-28.251	0.911	0.947
	N CT	IgG	4939 (13407)	5647 (14246.5)	4620.5 (12784.38)	-1026.5	0.308	0.504
		IgA	1295 (6068.5)	2062 (10124.75)	921.75 (3457.75)	-1140.25	0.009	0.031
		IgM	875.5 (1609)	888 (1256.25)	851.25 (2809.13)	-36.75	0.647	0.751
		IgG1	303 (3333)	427 (3048.5)	185 (4340)	-242	0.068	0.152
		IgG2	635.5 (410)	648 (369.5)	586.75 (374.25)	-61.25	0.293	0.502
		IgG3	147 (353)	223 (599.75)	110.75 (192.75)	-112.25	0.003	0.015
		IgG4	88.5 (9)	88.5 (8)	88.25 (9.5)	-0.25	0.892	0.947
	N FL	IgG	35320 (55351.5)	45965 (60962.75)	21934 (45309.75)	-24031	0.011	0.034
		IgA	20045 (48227)	22441 (52699)	17682 (35013.75)	-4759	0.249	0.448
		IgM	2321 (7310)	2216 (9539.75)	2370.5 (6097.63)	154.5	0.645	0.751
		IgG1	14359.5 (42843.5)	27729 (46870)	9557.75 (26749.25)	-18171.25	0.008	0.031
		IgG2	799 (1552)	984 (1449)	578.5 (1346)	-405.5	0.131	0.262
		IgG3	1167 (4479)	1695.5 (6009.25)	579.75 (2708.75)	-1115.75	0.003	0.015
		IgG4	79 (14.5)	80.5 (13)	78.75 (15.5)	-1.75	0.072	0.153
	**RBD**	**IgG**	**4607.5 (15308.5)**	**7150 (16723.75)**	**2073 (6634.38)**	**-5077**	**<0.001**	**<0.001**
		IgA	1894.5 (7515.5)	1201 (7014.5)	2693.25 (7480.75)	1492.25	0.736	0.828
		IgM	2757 (6090)	2570.5 (8663.5)	3117.25 (5069.75)	546.75	0.467	0.600
		**IgG1**	**412 (3792.5)**	**718 (5316.75)**	**142.5 (898.25)**	**-575.5**	**<0.001**	**0.003**
		IgG2	101 (26)	103 (29.5)	96.75 (14.75)	-6.25	0.023	0.060
		IgG3	111 (83)	119.5 (143.5)	97 (51.5)	-22.5	<0.001	0.003
		IgG4	80 (7.5)	80 (7.25)	80 (6.5)	0	0.964	0.964
	**S**	**IgG**	**11498 (27990.5)**	**16381 (31027.25)**	**8240 (14205.13)**	**-8141**	**0.001**	**0.005**
		IgA	4064.5 (11958.5)	4809.5 (14512.5)	4018.75 (10892.75)	-790.75	0.921	0.947
		IgM	3034 (5350)	2763.5 (4881.75)	3252.25 (5294.25)	488.75	0.436	0.581
		**IgG1**	**3028 (11894)**	**4533.5 (16100.5)**	**1364.5 (4825.75)**	**-3169**	**0.002**	**0.012**
		IgG2	86 (91)	95 (124.25)	78.25 (41.75)	-16.75	0.058	0.138
		**IgG3**	**166 (473.5)**	**258 (760.25)**	**101.5 (285.25)**	**-156.5**	**0.001**	**0.009**
		IgG4	54 (8.5)	54 (7.25)	53 (10.375)	-1	0.405	0.581
	**S2**	IgG	62231 (62093)	62231 (75538.75)	63158.25 (56209.25)	927.25	0.417	0.581
		IgA	23167 (39800.5)	26415.5 (52079.5)	21133.25 (32946.88)	-5282.25	0.209	0.395
		IgM	3464 (6485)	2492 (6549)	4507 (8222.5)	2015	0.009	0.031

		IgG1	44717 (57222)	41905 (81924.75)	46261.25 (47955.63)	4356.25	0.425	0.581
		IgG2	820 (1232)	836 (1226.75)	767 (1107.13)	-69	0.504	0.626
		**IgG3**	**940 (3937)**	**1185 (5613.75)**	**657.5 (2378.25)**	**-527.5**	**0.016**	**0.044**
		IgG4	86.5 (18)	86.5 (19)	85.75 (17.5)	-0.75	0.408	0.581
	N CT	IgG AI	0.34 (0.30)	0.333 (0.259)	0.4 (0.356)	0.067	0,815335	0,815
	N FL		0.39 (0.24)	0.408 (0.211)	0.343 (0.299)	-0.065	0,138256	0,173
	RBD		**0.20 (0.21)**	**0.27 (0.21)**	**0.11 (0.14)**	**-0.157**	**<0.001**	**<0.001**
	S		**0.18 (0.19)**	**0.25 (0.19)**	**0.10 (0.11)**	**-0.152**	**<0.001**	**<0.001**
	S2		**0.64 (0.25)**	**0.67 (0.28)**	**0.60 (0.26)**	**-0.070**	**0,027**	**0,045**
Day 60	ID50	ID50	821.04 (1522.50)	861.525 (2311.61)	810.08 (1145.90)	-51.45	0.394	0.835
	N CT	IgG	6408 (7720)	6035 (7587.5)	7148 (7643.88)	1113	0.291	0.785
		IgA	403.5 (719.5)	375 (882.5)	424.75 (627.25)	49.75	0.763	0.924
		IgM	687 (922.5)	737 (883.25)	618.25 (963.25)	-118.75	0.372	0.835
		IgG1	753 (1970)	506.5 (1791.25)	1044.25 (2449.75)	537.75	0.144	0.616
		IgG2	686 (493)	695 (472.25)	681.5 (567.63)	-13.5	0.755	0.924
		IgG3	209.5 (364.5)	209.5 (250.5)	210.75 (421.38)	1.25	0.757	0.924
		IgG4	87 (7)	89 (7.75)	86 (6)	-3	0.037	0.457
	N FL	IgG	56646 (48193.5)	57289 (44839.5)	56178.5 (47733.25)	-1110.5	0.964	0.974
		IgA	3297 (9064)	3388.5 (10667.5)	3295.25 (5304.25)	-93.25	0.643	0.924
		IgM	1578 (2660)	1492.5 (2839)	1673.75 (2485.25)	181.25	0.245	0.785
		IgG1	46192 (51563)	43587.5 (52749.5)	49511.25 (49738.63)	5923.75	0.774	0.924
		IgG2	2224 (4868.5)	2395 (4238.5)	1968 (5554)	-427	0.974	0.974
		IgG3	3284.5 (7915.5)	3137 (5867.5)	4526.25 (8723.88)	1389.25	0.552	0.924
		IgG4	87 (30.5)	89 (28.5)	84 (25.38)	-5	0.122	0.616
	RBD	IgG	19096.5 (33599.5)	21565 (39970)	16576.5 (28807.38)	-4988.5	0.146	0.616
		IgA	1718.5 (4582)	1718.5 (5177.5)	1786 (3672.38)	67.5	0.586	0.924
		IgM	2491.5 (6469.5)	2624 (6622.5)	2273 (6297)	-351	0.847	0.924
		IgG1	6194.5 (20052)	7057.5 (24823)	5425.5 (14106)	-1632	0.294	0.785
		IgG2	118 (93)	124 (115)	113.25 (66.88)	-10.75	0.327	0.785
		IgG3	247 (589)	297 (917.5)	213 (490.25)	-84	0.038	0.457
		IgG4	82 (6)	82 (8.25)	82 (5.38)	0	0.529	0.924
	S	IgG	27083.5 (38393)	29756 (45595)	24332 (34887.5)	-5424	0.110	0.616
		IgA	2649 (5549)	2605 (6525.5)	2740.5 (4207.38)	135.5	0.699	0.924
		IgM	2076 (3956)	1858 (5033.75)	2226.25 (3293.13)	368.25	0.768	0.924

		IgG1	13719 (31290)	14915 (33775)	13207 (25167.25)	-1708	0.308	0.785
		IgG2	165 (412)	139.5 (500)	190.75 (383.75)	51.25	0.900	0.952
		IgG3	645 (1672.5)	761 (2869.75)	571 (1167.75)	-190	0.154	0.616
		IgG4	54.5 (7.5)	57 (8.5)	53 (6)	-4	<0.001	0.007
	S2	IgG	80433 (57093)	79430 (55653)	80678.5 (59774.75)	1248.5	0.839	0.924
		IgA	16149 (22778)	17475.5 (26600)	12760.25 (19330.88)	-4715.25	0.468	0.924
		IgM	2066 (3423)	1880 (3784.5)	2472.5 (2662.88)	592.5	0.190	0.682
		IgG1	69530.5 (58673.5)	71248 (60644.5)	68314.5 (57521.38)	-2933.5	0.645	0.924
		IgG2	1230.5 (1811)	1229 (2050.75)	1264.75 (1703)	35.75	0.520	0.924
		IgG3	3692.5 (8878)	3692.5 (13081.75)	3689 (7458.5)	-3.5	0.820	0.924
		IgG4	88 (16)	91 (18.5)	86.25 (13)	-4.75	0.137	0.616
	N CT	IgG AI	0.32 (0.35)	0.39 (0.33)	0.30 (0.32)	-0.09	0.796	0.800
	N FL		0.66 (0.17)	0.65 (0.17)	0.66 (0.18)	0.01	0.608	0.759
	RBD		0.46 (0.23)	0.48 (0.24)	0.45 (0.20)	-0.03	0.040	0.115
			0.43 (0.20)	0.45 (0.22)	0.38 (0.19)	-0.07	0.046	0.115
	S		0.69 (0.18)	0.70 (0.18)	0.68 (0.19)	-0.02	0.141	0.235
	S2		0.32 (0.35)	0.40 (0.33)	0.30 (0.32)	-0.09	0.796	0.796

Legend: Table showing levels of antibodies in median (IQR) in study participants – overall, MB-CCP group and placebo group; and difference in median between placebo and MB-CCP groups and *p-values* at different timepoints – baseline, day 7 and day 60 after infusion. We show *p-values* unadjusted and adjusted by timepoints. Marked in bold are differences statistically significant (*p*<0.05) and above the seropositivity cutoffs defined by levels > mean+3SD of 92 and 128 (IgA/IgM and IgG, respectively) prepandemic controls (ISGlobal).

Ag: Antigen; Ab: Antibody; MB-CPP group: Methylene blue-treated COVID-19 convalescent plasma group; ID50: 50% inhibitory dilution; N CT: nucleocapsid C-terminal region; N FL: nucleocapsid full length protein; RBD: receptor binding domain; S: spike full protein; S2: S2 fragment; Adj p-value: adjusted p-value; AI: avidity index.

Table S2: Increase in levels of antibodies between baseline and day 7 and day 60 in the overall population

Ag	Abs	Increase baseline – day 7	Increase baseline – day 60	Increase day 7 – day 60
	**IgG**	5.27 (16.94)	3.42 (4.07)	1.35 (1.29)
	**IgA**	32.97 (87.16)	5.3 (24.33)	0.73 (1.81)
	**IgM**	4.84 (16.73)	1.52 (1.73)	0.91 (0.86)
	**IgG1**	18.22 (40.77)	13.2 (33.48)	3.49 (6.86)
	**IgG2**	2.13 (6.04)	3.33 (9.93)	2.15 (6.18)
	**IgG3**	12.95 (65.19)	6.74 (25.39)	2.76 (8.32)
**N CT**	**IgG4**	1.03 (0.13)	1.03 (0.16)	1 (0.14)
	**IgG**	14.3 (20.11)	22.07 (25.3)	2.8 (4.42)
	**IgA**	49.14 (83.94)	10.1 (18.93)	0.5 (0.9)
	**IgM**	5.58 (10.26)	2.99 (5.55)	0.91 (0.96)
	**IgG1**	58.43 (87.5)	124.83 (131.21)	9.72 (32.94)
	**IgG2**	4.6 (11.56)	10.98 (19.55)	4.1 (7.61)
	**IgG3**	21.65 (45.64)	40.54 (51.48)	10.62 (26.41)
**N FL**	**IgG4**	2.2 (8.67)	1.48 (1.07)	1.21 (0.58)
	**IgG**	42.28 (71.08)	111.05 (126.5)	12.76 (40.17)
	**IgA**	27.17 (47.54)	26.96 (73.83)	4.61 (13.77)
	**IgM**	10.95 (15.16)	15.7 (45.61)	4.12 (22.41)
	**IgG1**	39.22 (82.28)	140.26 (203.5)	40.07 (107.18)
	**IgG2**	1.3 (1.08)	4.64 (15.75)	3.21 (10.63)
	**IgG3**	5.86 (29.73)	9.88 (25.26)	6.39 (20.76)
**RBD**	**IgG4**	1 (0.09)	1.03 (0.11)	1.03 (0.1)
	**IgG**	49.86 (71.84)	114.27 (155.01)	5.97 (20.33)
	**IgA**	41.41 (62.21)	41.74 (102.53)	3.48 (11.35)
	**IgM**	11.13 (13.92)	15.48 (39.25)	3.65 (17.46)
	**IgG1**	99.8 (175.23)	299.89 (403.48)	40.06 (177.8)
	**IgG2**	4.31 (9.58)	10.86 (28.87)	5.32 (20.71)
	**IgG3**	10.9 (25.24)	34.37 (105.99)	16.63 (74.41)
**S**	**IgG4**	1.03 (0.16)	1.07 (0.23)	1.05 (0.24)
	**IgG**	18.87 (21.69)	25.23 (26.47)	1.92 (3.03)
	**IgA**	41.42 (55.31)	32.2 (61.39)	1.25 (2.12)
	**IgM**	8.7 (11.18)	5.8 (8.45)	1.24 (2.74)
	**IgG1**	153.92 (212.15)	232.72 (270.14)	9.24 (51.57)
	**IgG2**	5.09 (15.36)	6.09 (16.77)	1.79 (2.09)
	**IgG3**	19.11 (39.13)	45.54 (104.56)	11.58 (46.66)
**S2**	**IgG4**	1.1 (0.28)	1.12 (0.25)	1.04 (0.23)
**nAbs**	**ID_{50}**	15.42 (18.46)	22.18 (43.6)	3.39 (10.82)

Legend: Table showing increase, in mean (SD), of antibody isotypes and subclasses, as well as neutralizing antibodies between baseline and day 7, baseline and day 60, and day 7 and day 60, in the overall study population.

Figure S2: Increase in antibody levels from baseline to day 7 in overall study participants (MB-CCP and placebo groups) stratified by serostatus of participants at baseline

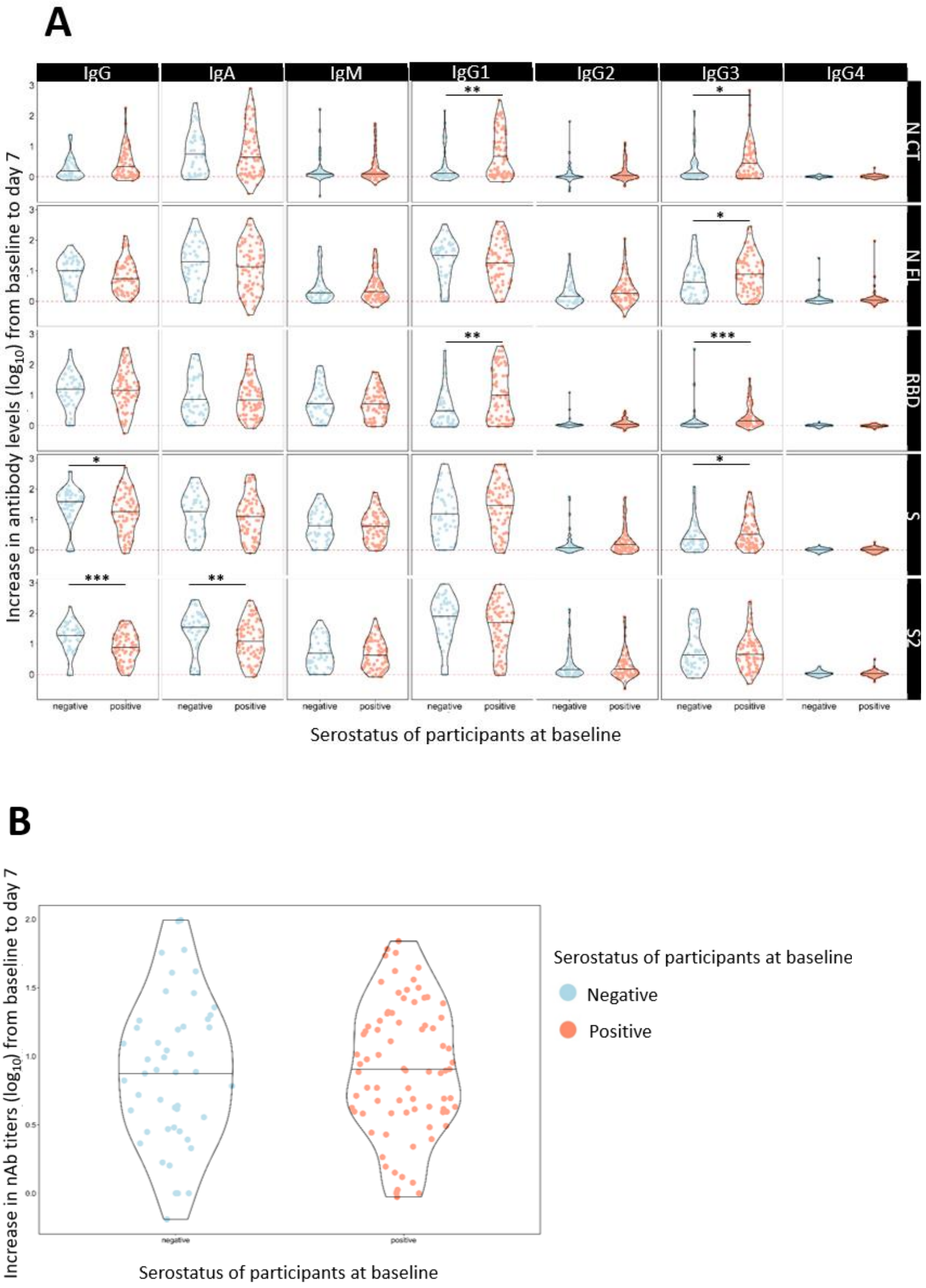

Legend: Violin plots showing increase in antibody levels (in $\log_{10}$) from baseline to day 7 in overall study participants (MB-CCP and placebo groups), stratified according to serostatus of participants at baseline (negative and positive). Baseline serostatus was defined by Luminex as antibody levels > mean+3SD of negative prepandemic controls: Seronegative = IgG negative (IgG1, IgG2, IgG3, IgG4) + IgM negative + IgA negative; Seropositive = any positive response for any of the isotypes/antigen pairs. Median showing as a thick solid line. **A.** Antibody isotypes (IgG, IgM, IgA) and subclasses (IgG1, IgG2, IgG3, IgG4) ($\log_{10}$ MFI). **B.** Neutralizing antibody titers ($\log_{10}$ ID50).

Asterisks are indicating statistically significant (*p <0.05, ** p <0.01, ***p <0.001) differences in increments estimated with antibody levels above the seropositivity cutoffs.

Figure S3: Increase in antibody levels from baseline to day 7 in participants from MB-CCP group stratified by serostatus of participants at baseline

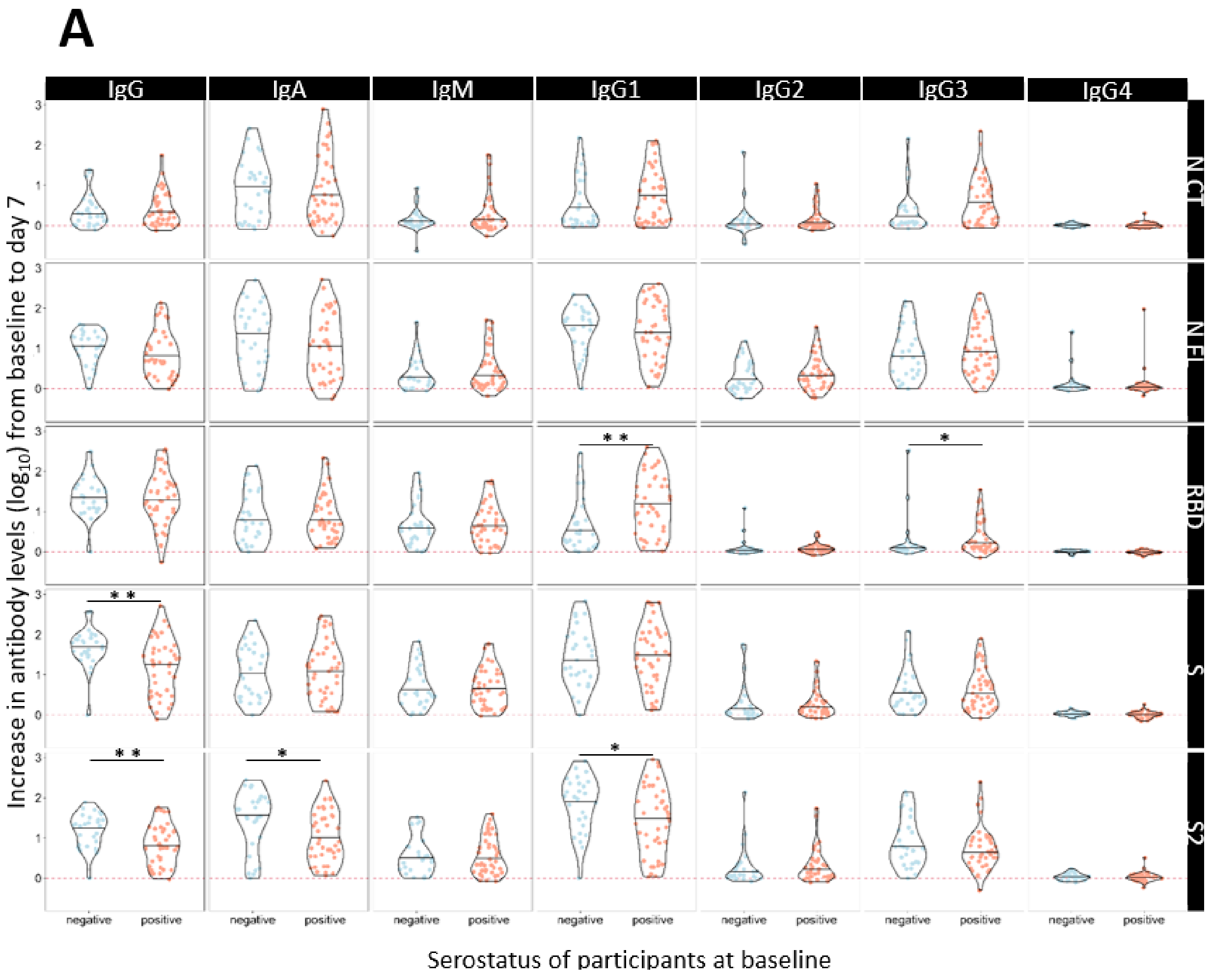

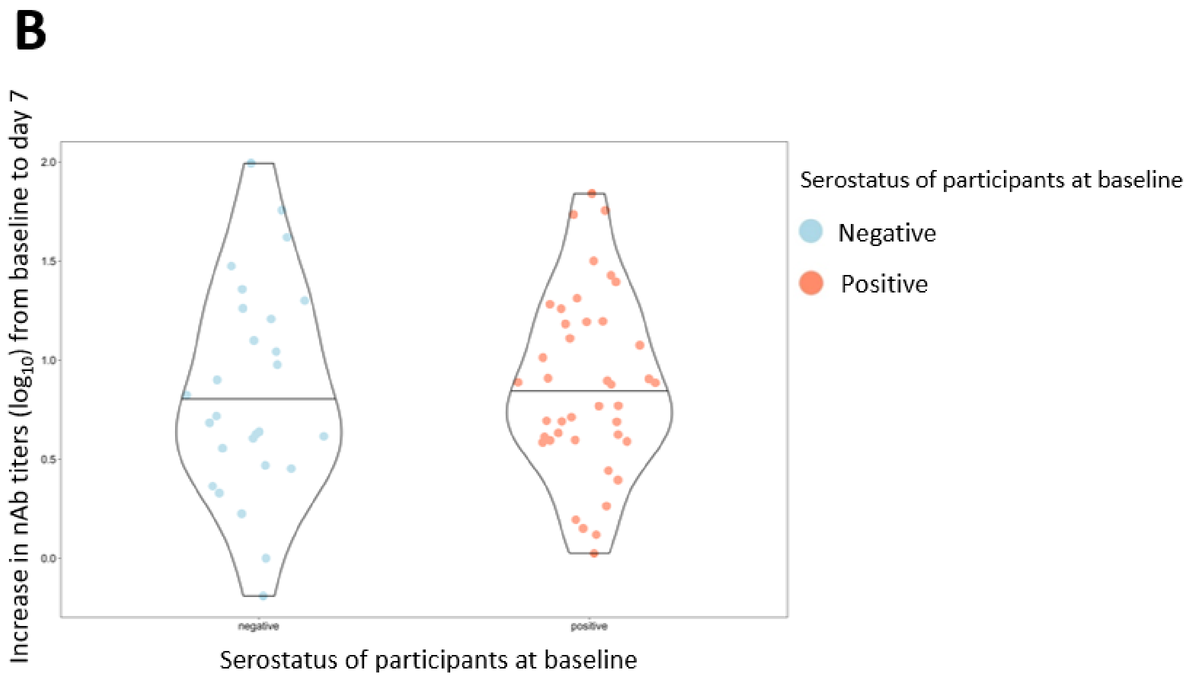

<u>Legend</u>: Violin plots showing the increase in antibody levels (in $\log_{10}$) from baseline to day 7 in study participants who received MB-CCP infusion, stratified according to serostatus of participants at baseline (negative and positive). Baseline serostatus was defined by Luminex as antibody levels > mean+3SD of negative prepandemic controls: Seronegative = IgG negative (IgG1, IgG2, IgG3, IgG4) + IgM negative + IgA negative; Seropositive = any positive response for any of the isotypes/antigen pairs. Median showing as a thick solid line. **A.** Antibody isotypes (IgG, IgM, IgA) and subclasses (IgG1, IgG2, IgG3, IgG4) ($\log_{10}$ MFI). **B.** Neutralizing antibody titers ($\log_{10}$ ID50).

Asterisks are indicating statistically significant (*p <0.05, ** p <0.01, ***p <0.001) differences in increments estimated with antibody levels above the seropositivity cutoffs.

Figure S4: Increase in antibody levels from baseline to day 7 in participants from MB-CCP group stratified by neutralizing activity of convalescent plasma infused

A

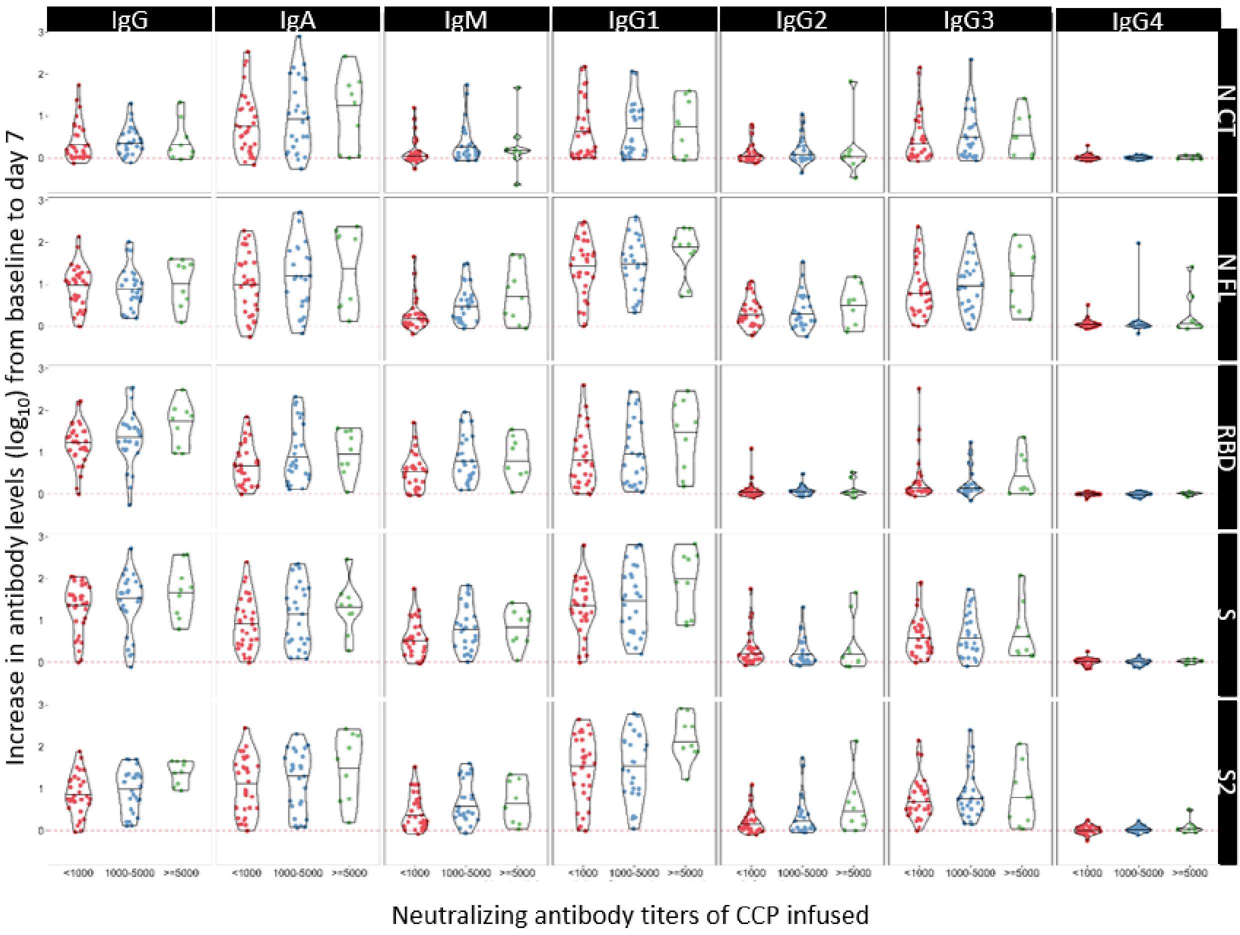

B

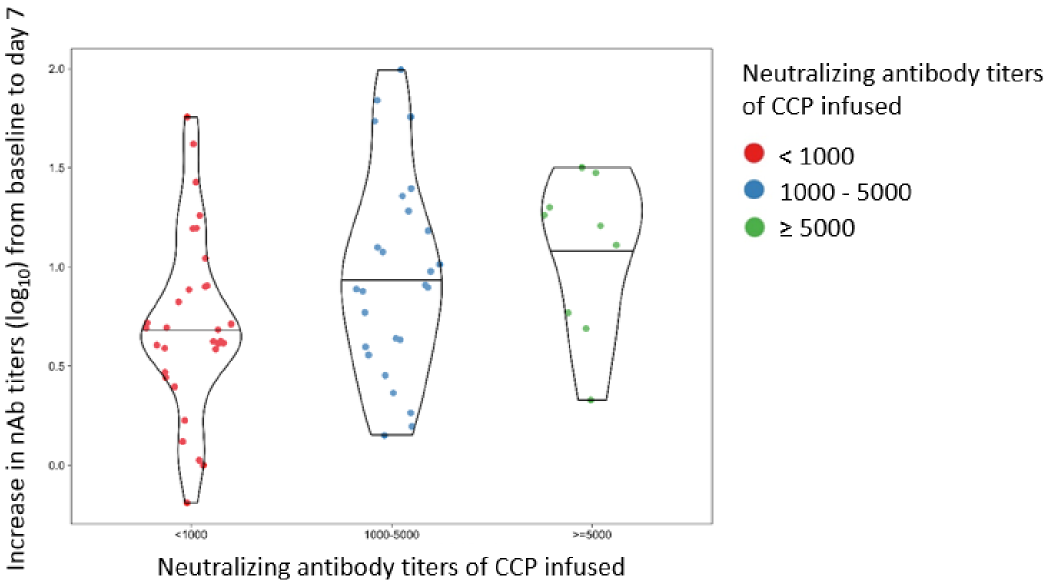

<u>Legend</u>: Violin plots showing the increase in antibody levels (in $\log_{10}$) from baseline to day 7 in study participants who received MB-CCP infusion, stratified according to neutralizing antibody titers of MB-CCP infused (ID50 <1000, 1000 – 5000, ≥5000). Neutralizing antibody titers (ID50) were assessed by pseudoviral neutralization assay against Wuhan/WH1. Median showing as a thick solid line. **A.** Antibody isotypes (IgG, IgM, IgA) and subclasses (IgG1, IgG2, IgG3, IgG4) ($\log_{10}$ MFI). **B.** Neutralizing antibody titers ($\log_{10}$ ID50).

Asterisks are indicating statistically significant (**p* <0.05, ** *p* <0.01, ****p* <0.001) differences in increments estimated with antibody levels above the seropositivity cutoffs.

Table S3: Distribution of anti-SARS-CoV-2 antibody levels in methylene blue-treated COVID-19 convalescent plasma (MB-CCP) units and in participants that received MB-CCP infusion (MB-CCP group) at day 7 after infusion

Ag	Ab	MB-treated CCP units (donors)	MB-CCP group at day 7	MB-CCP group day 7 – CCP units	*p*-value	Adjusted *p*-value
WH1	ID50	1207 (2169)	1017.1 (2029.35)	-189.9	0.646	0.646
N CT	IgG	5999 (6217.63)	5647 (14246.50)	-352	0.473	0.473
	IgA	262.5 (311.75)	2062 (10124.75)	1799.5	<0.001	<0.001
	IgM	386 (350)	888 (1256.25)	502	<0.001	<0.001
	IgG1	1270.5 (4759.88)	427 (3048.50)	-843.5	0.002	0.003
	IgG2	413.5 (489.5)	648 (369.50)	234.5	0.004	0.006
	IgG3	168.5 (148.38)	223 (599.75)	54.50	0.457	0.473
	IgG4	99.25 (18.88)	88.5 (8.00)	-10.75	<0.001	<0.001
N FL	IgG	58250.75 (39223.75)	45965 (60962.75)	-12285.75	0,022	0,025
	IgA	1772.75 (2881.75)	22441 (52699)	20668.25	<0.001	<0.001
	IgM	879 (1185.38)	2216 (9539.75)	1337	<0.001	<0.001
	IgG1	61278.5 (42530.88)	27729 (46870)	-33549.50	<0.001	<0.001
	IgG2	1356.75 (3090)	984 (1449)	-372.75	0.021	0.025
	IgG3	1804.5 (4488.25)	1695.5 (6009.25)	-109	0.660	0.660
	IgG4	210.5 (366.625)	80.5 (13)	-130	<0.001	<0.001
RBD	IgG	34423 (22347)	7150 (16723.75)	-27273	<0.001	<0.001
	IgA	1764.25 (3266.5)	1201 (7014.5)	-563.25	0.319	0.319
	IgM	2555.5 (4232.125)	2570.5 (8663.5)	15	0.227	0.259
	IgG1	21978.5 (15707)	718 (5316.75)	-21260.5	<0.001	<0.001
	IgG2	193 (133)	103 (29.5)	-90	<0.001	<0.001
	IgG3	273 (260.75)	119.5 (143.5)	-153.5	<0.001	<0.001
	IgG4	95.5 (16)	80 (7.25)	-15.5	<0.001	<0.001
S	IgG	42937 (18293.38)	16381 (31027.25)	-26556	<0.001	<0.001
	IgA	3425 (5429.5)	4809.5 (14512.5)	1384.5	0.742	0.742
	IgM	2492.75 (3527.5)	2763.5 (4881.75)	270.75	0.322	0.368
	IgG1	35387.75 (16138.25)	4533.5 (16100.5)	-30854.25	<0.001	<0.001
	IgG2	201 (157.125)	95 (124.25)	-106	<0.001	<0.001
	IgG3	486.75 (765)	258 (760.25)	-228.75	0.002	0.002
	IgG4	68.75 (26.75)	54 (7.25)	-14.75	<0.001	<0.001
S2	IgG	83647.25 (30737.88)	62231 (75538.75)	-21416.25	0.005	0.007
	IgA	15661.5 (14947.88)	26415.5 (52079.5)	10754	0,001	0,001
	IgM	1162.5 (2123.625)	2492 (6549)	1329.5	<0.001	<0.001
	IgG1	97449.75 (38580)	41905 (81924.75)	-55544.75	<0.001	<0.001
	IgG2	972 (1234.75)	836 (1226.75)	-136	0.601	0.601
	IgG3	2914.75 (2907.25)	1185 (5613.75)	-1729.75	0.005	0.007
	IgG4	125.75 (52.125)	86.5 (19)	-39.25	<0.001	<0.001
N CT	IgG avidity Index	0.462 (0.126)	0.333 (0.259)	-0.129	0.424	0.473
N FL		0.681 (0.258)	0.408 (0.211)	-0.273	<0.001	<0.001
RBD		0.616 (0.291)	0.265 (0.211)	-0.351	<0.001	<0.001
S		0.584 (0.288)	0.250 (0.188)	-0.334	<0.001	<0.001
S2		0.746 (0.189)	0.671 (0.282)	-0.075	0.026	0.030

Legend: Table showing difference, in median (IQR), of antibody isotypes and subclasses, as well as neutralizing antibodies and IgG avidity index between MB-treated CCP units (donors) and participants that received MB-CCP infusion (MB-CCP group) at day 7 after infusion.

Figure S5: Correlation between neutralizing antibody titers and antibody levels in MB-treated CCP units (donors)

<u>Legend</u>: Correlations of neutralizing antibody titers (in log_{10} ID_{50}) and antibody levels (in log_{10} MFI) in CCP units (donors). Antibody isotypes (IgG, IgM, IgA) and subclasses (IgG1, IgG2, IgG3, IgG4) were measured by Luminex. Neutralizing antibody titers (ID_{50}) were assessed by pseudoviral neutralization assay against Wuhan/WH1.

ρ (rho) and *p*-values were calculated by Spearman and are indicated in the figure.

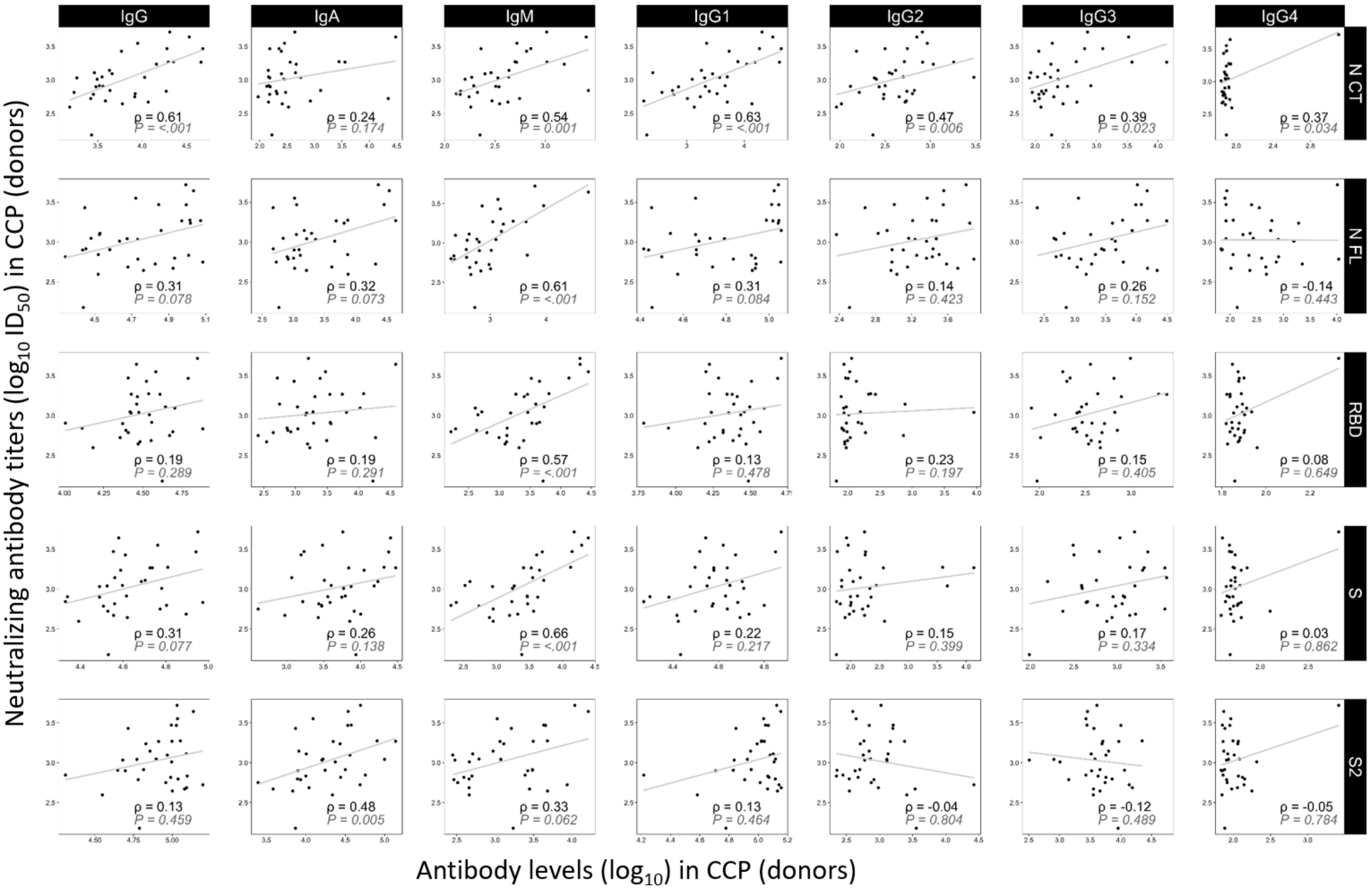

IgG
IgA
IgM
IgG1
IgG2
IgG3
IgG4
N CT
N FL
RBD
S
S2
ρ = 0.61
P = <.001
ρ = 0.24
P = 0.174
ρ = 0.54
P = 0.001
ρ = 0.63
P = <.001
ρ = 0.47
P = 0.006
ρ = 0.39
P = 0.023
ρ = 0.37
P = 0.034
ρ = 0.31
P = 0.078
ρ = 0.32
P = 0.073
ρ = 0.61
P = <.001
ρ = 0.31
P = 0.084
ρ = 0.14
P = 0.423
ρ = 0.26
P = 0.152
ρ = -0.14
P = 0.443
ρ = 0.19
P = 0.289
ρ = 0.19
P = 0.291
ρ = 0.57
P = <.001
ρ = 0.13
P = 0.478
ρ = 0.23
P = 0.197
ρ = 0.15
P = 0.405
ρ = 0.08
P = 0.649
ρ = 0.31
P = 0.077
ρ = 0.26
P = 0.138
ρ = 0.66
P = <.001
ρ = 0.22
P = 0.217
ρ = 0.15
P = 0.399
ρ = 0.17
P = 0.334
ρ = 0.03
P = 0.862
ρ = 0.13
P = 0.459
ρ = 0.48
P = 0.005
ρ = 0.33
P = 0.062
ρ = 0.13
P = 0.464
ρ = -0.04
P = 0.804
ρ = -0.12
P = 0.489
ρ = -0.05
P = 0.784
Neutralizing antibody titers (log10 ID50) in CCP (donors)
Antibody levels (log10) in CCP (donors)

Figure S6. Correlation between antibody levels in MB-CCP units (from donors) and in participants treated with MB-CCP at day 7.

A

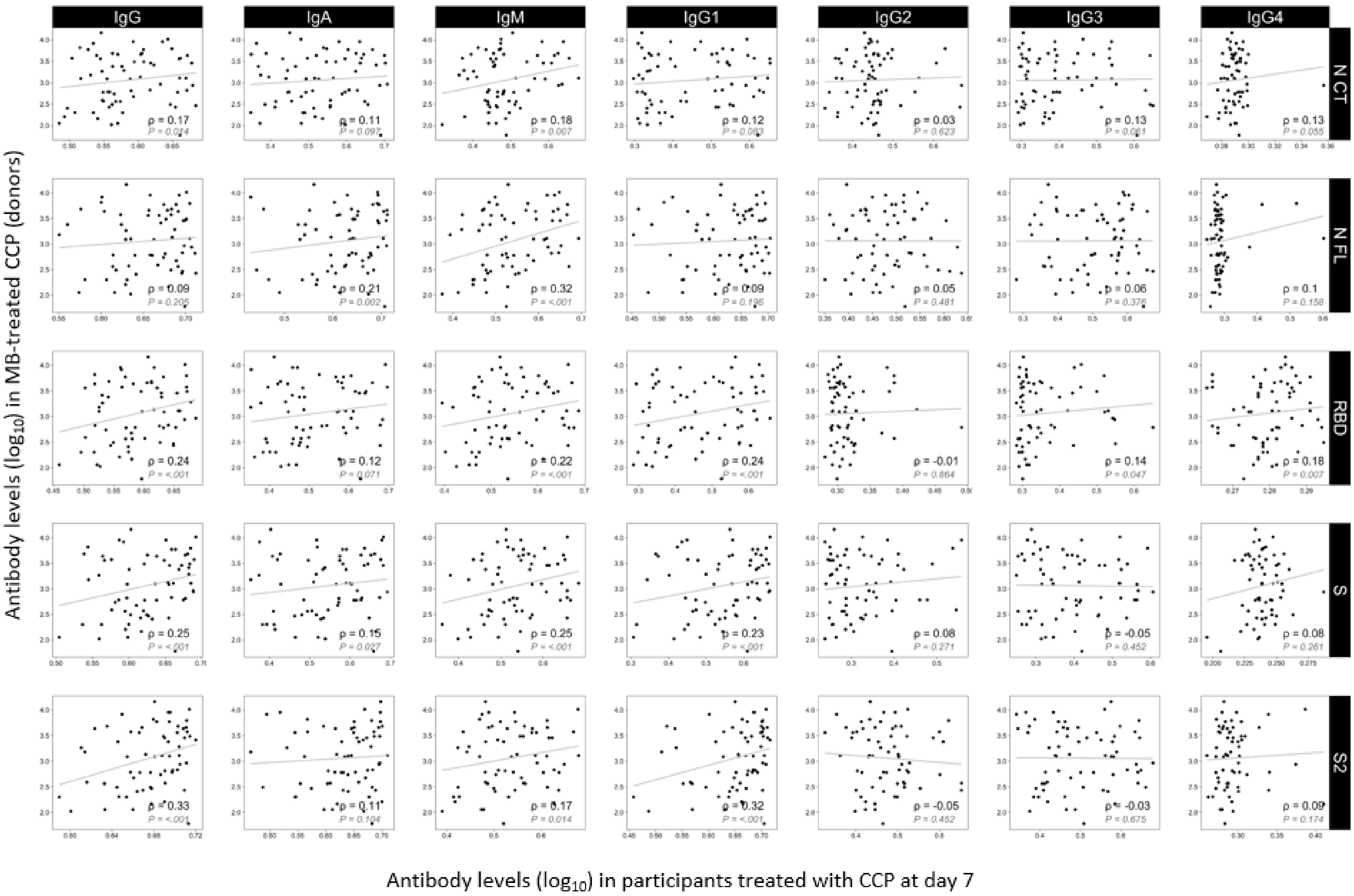
IgG
IgA
IgM
IgG1
IgG2
IgG3
IgG4
N CT
N FL
RBD
S
S2
ρ = 0.17
ρ = 0.11
ρ = 0.18
ρ = 0.12
ρ = 0.03
ρ = 0.13
ρ = 0.13
ρ = 0.09
ρ = 0.21
ρ = 0.32
ρ = 0.09
ρ = 0.05
ρ = 0.06
ρ = 0.1
ρ = 0.24
ρ = 0.12
ρ = 0.22
ρ = 0.24
ρ = -0.01
ρ = 0.14
ρ = 0.18
ρ = 0.25
ρ = 0.15
ρ = 0.25
ρ = 0.23
ρ = 0.08
ρ = -0.05
ρ = 0.08
ρ = 0.33
ρ = 0.11
ρ = 0.17
ρ = 0.32
ρ = -0.05
ρ = -0.03
ρ = 0.09
Antibody levels (log_{10}) in MB-treated CCP (donors)
Antibody levels (log_{10}) in participants treated with CCP at day 7

B

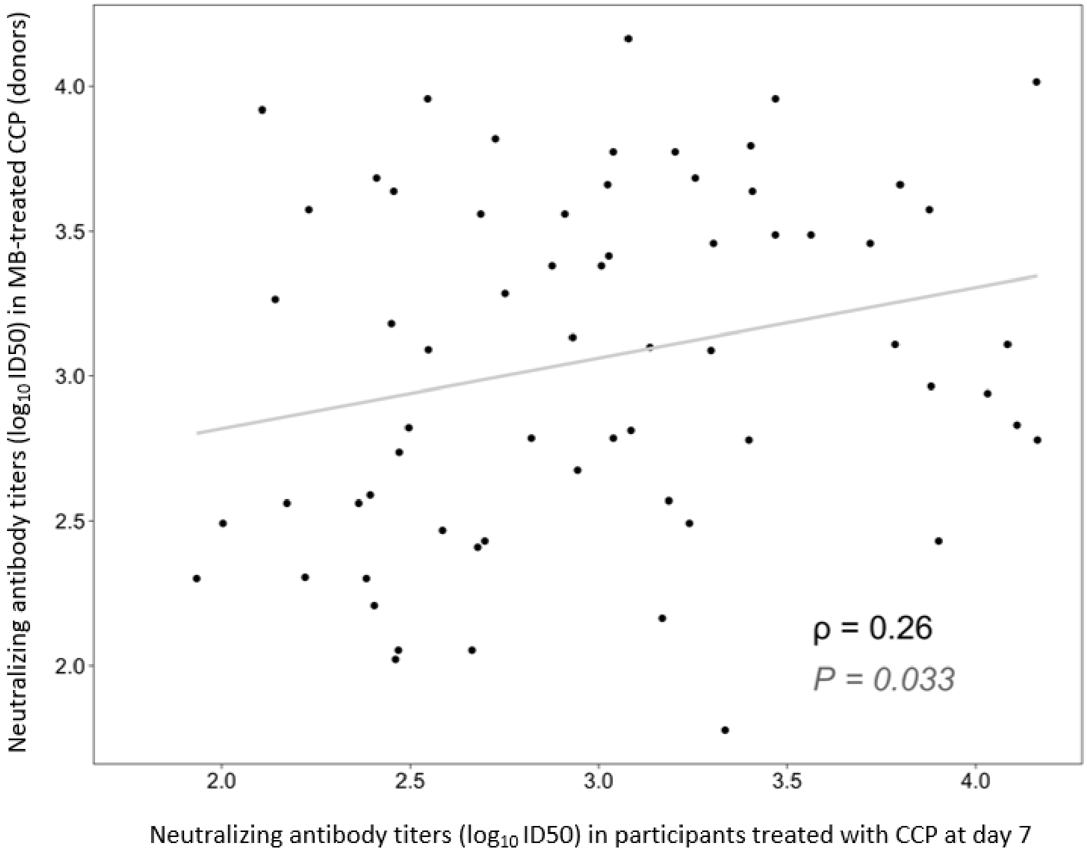

Legend: Correlations of antibody levels (log_{10} MFI and ID_{50}) in methylene blue-treated COVID-19 convalescent plasma (MB-CCP) (donors) (Y axis) and participants treated with MB-CCP at day 7 (X axis). **A.** Correlation of antibody isotypes (IgG, IgM, IgA) and subclasses (IgG1, IgG2, IgG3, IgG4) (in log_{10} MFI) measured by Luminex. **B.** Correlation of neutralizing antibody titers (log_{10} ID50) assessed by pseudoviral neutralization assay against Wuhan/WH1.

ρ (rho) and *p*-values were calculated by Spearman and are indicated in the figure.

Figure S7: Correlation between the increase of neutralizing antibody titers and antibody levels from baseline to day 7 in participants treated with MB-CCP

Legend: Correlations of the increase of neutralizing antibody titers (in log_{10} ID_{50}) and increase of antibody levels (in log_{10} MFI) from baseline to day 7 in participants treated with MB-CCP. Antibody isotypes (IgG, IgM, IgA) and subclasses (IgG1, IgG2, IgG3, IgG4) were measured by Luminex. Neutralizing antibody titers (ID_{50}) were assessed by pseudoviral neutralization assay against Wuhan/WH1.

ρ (rho) and *p*-values were calculated by Spearman and are indicated in the figure.

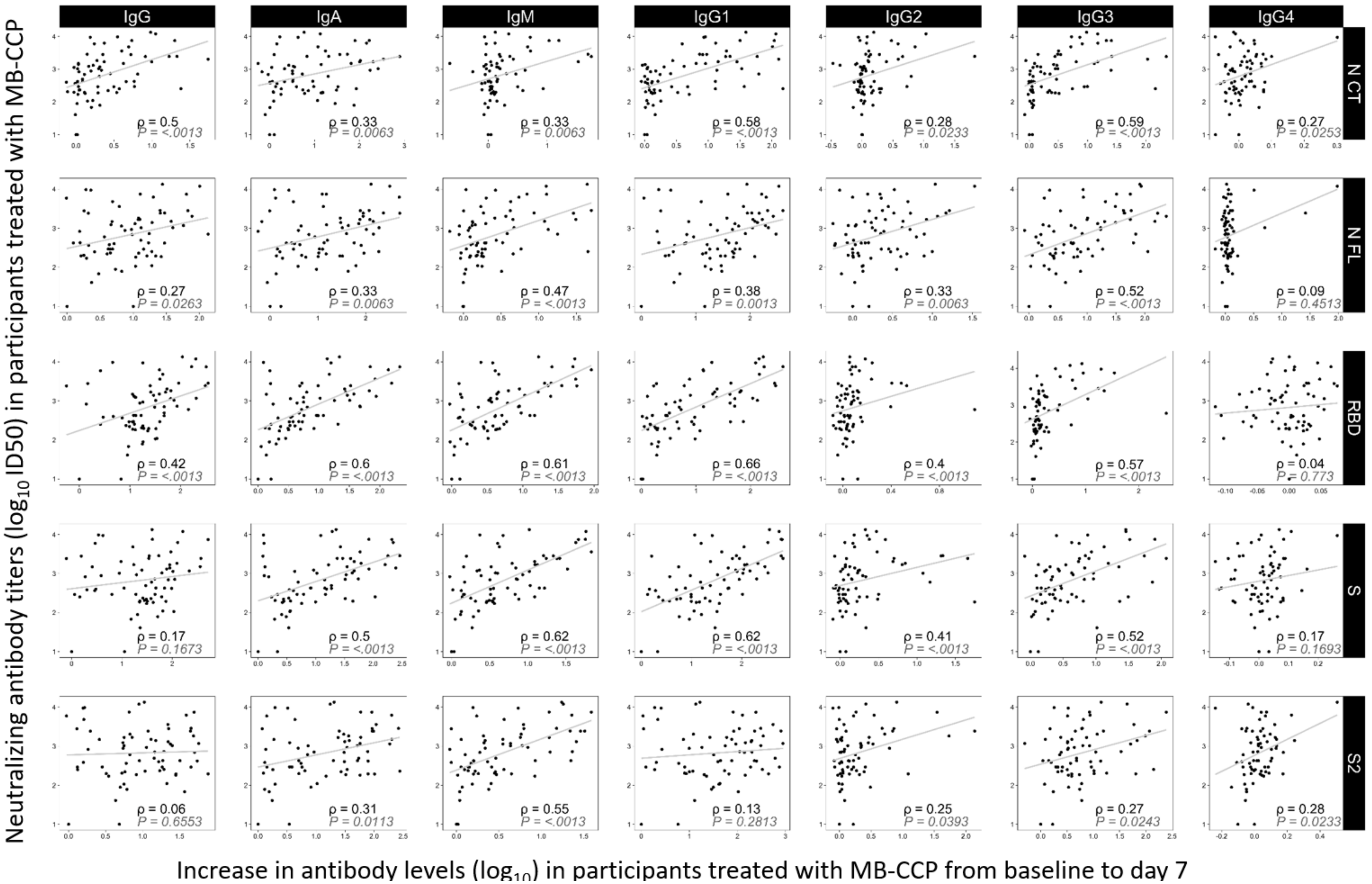
IgG
IgA
IgM
IgG1
IgG2
IgG3
IgG4
N CT
N FL
RBD
S
S2
ρ = 0.5 P = <.0013
ρ = 0.33 P = 0.0063
ρ = 0.33 P = 0.0063
ρ = 0.58 P = <.0013
ρ = 0.28 P = 0.0233
ρ = 0.59 P = <.0013
ρ = 0.27 P = 0.0253
ρ = 0.27 P = 0.0263
ρ = 0.33 P = 0.0063
ρ = 0.47 P = <.0013
ρ = 0.38 P = 0.0013
ρ = 0.33 P = 0.0063
ρ = 0.52 P = <.0013
ρ = 0.09 P = 0.4513
ρ = 0.42 P = <.0013
ρ = 0.6 P = <.0013
ρ = 0.61 P = <.0013
ρ = 0.66 P = <.0013
ρ = 0.4 P = <.0013
ρ = 0.57 P = <.0013
ρ = 0.04 P = 0.773
ρ = 0.17 P = 0.1673
ρ = 0.5 P = <.0013
ρ = 0.62 P = <.0013
ρ = 0.62 P = <.0013
ρ = 0.41 P = <.0013
ρ = 0.52 P = <.0013
ρ = 0.17 P = 0.1693
ρ = 0.06 P = 0.6553
ρ = 0.31 P = 0.0113
ρ = 0.55 P = <.0013
ρ = 0.13 P = 0.2813
ρ = 0.25 P = 0.0393
ρ = 0.27 P = 0.0243
ρ = 0.28 P = 0.0233
Neutralizing antibody titers (log_{10} ID50) in participants treated with MB-CCP
Increase in antibody levels (log_{10}) in participants treated with MB-CCP from baseline to day 7

Table S4: Effect of Methylene Blue treatment on levels of SARS-CoV-2 neutralizing antibodies, isotypes and subclasses for COVID-19 convalescent plasma.

Ag	Ab	Pre-MB Median (IQR) (n=40)	Post-MB median_(IQR) (n=40)	Difference in median post-pre MB treatment	*p-values*
	IgG	5856.63 (8111.92)	6062 (5986.75)	205.37	0.077
	IgA	226.66 (246.63)	230 (295.50)	3.34	0.875
	IgM	341.29 (316.54)	295 (301.25)	-46.29	0.637
	IgG1	2211.87 (5906.35)	1493 (4326.50)	-718.87	0.430
	IgG2	414.24 (451.14)	361.50 (435.50)	-52.74	0.637
	IgG3	185.41 (194.96)	170.50 (128)	-14.91	0.430
N CT	IgG4	78.16 (7.82)	99.50 (19.75)	21.34	<0.001
	IgG	54172.97 (51066.41)	60411 (43416)	6238.03	0.637
	IgA	1620.47 (4792.16)	1285 (3376.75)	-335.47	<0.001
	IgM	824.57 (1028.43)	735 (1185)	-89.57	0.875
	IgG1	62083.42 (54230.94)	62730.50 (47448.50)	647.08	0.268
	IgG2	1735.11 (2013.87)	1308 (2008)	-427.11	0.430
	IgG3	2713.82 (6517.07)	1886 (4171.50)	-827.82	<0.001
N FL	IgG4	252.28 (676.50)	212 (410.25)	-40.28	0.430
	IgG	33193.66 (15016.30)	32843.50 (14815)	-350.16	0.875
	**IgA**	**1890.55 (3086.80)**	**1551 (3064.50)**	**-339.55**	**<0.001**
	IgM	2482.82 (3436.56)	2450 (4132.50)	-32.82	0.036
	IgG1	24180.75 (13942.07)	20192 (12403.25)	-3988.75	0.078
	**IgG2**	**103.34 (72.30)**	**179 (86)**	**75.66**	**<0.001**
	IgG3	326.53 (271.16)	276 (277.25)	-50.53	0.637
**RBD**	IgG4	75.55 (7.38)	94 (16)	18.45	<0.001
	IgG	41247.39 (24975.57)	42877 (18293.50)	1629.61	0.430
	**IgA**	**5775 (5612.39)**	**3726 (4077)**	**-2049.00**	**<0.001**
	IgM	2935.26 (3847.76)	2753.50 (3152.25)	-181.76	0.430
	**IgG1**	**41685.51 (18510.83)**	**35485.50 (15114.75)**	**-6200.01**	**0,001**
	**IgG2**	**130.70 (112.24)**	**187.50 (117.50)**	**56.80**	**<0.001**
**S**	**IgG3**	**870.16 (1100.07)**	**560 (707.75)**	**-310.16**	**<0.001**
	IgG4	52.11 (8.68)	69.50 (23.75)	17.40	<0.001
	IgG	97871.05 (42147.73)	85685.50 (26754.25)	-12185.55	0.268
	**IgA**	**19147.82 (24518.78)**	**14868.50 (14575.75)**	**-4279.32**	**<0.001**
	IgM	1296.55 (2617.64)	1178 (2276.75)	-118.55	<0.001
	**IgG1**	**109843.97 (38433.06)**	**93975 (42124)**	**-15868.97**	**<0.001**
	IgG2	787.22 (1082.49)	748.50 (899.75)	-38.72	0.152
	**IgG3**	**4587 (5851.64)**	**2858 (2454.50)**	**-1729.00**	**<0.001**
**S2**	IgG4	92.92 (37.99)	126 (53.75)	33.08	<0.001
WH1	ID50	1052.96 (1226.43)	1191.50 (2377.50	138.54	0.077

Legend: Table showing levels of antibodies in median (IQR) in COVID-19 convalescent plasma before and after treatment with methylene blue; and difference of median post-pre methylene blue treatment, with *p-values*. Marked in bold are differences statistically significant (p<0.05) and above the seropositivity cutoffs defined as the mean+3SD of 92 and 128 (IgA/IgM and IgG, respectively) prepandemic controls (ISGlobal).

Ag: Antigen; Ab: Antibody; MB: Methylene blue; pre-MB: before methylene blue treatment; post-MB: after methylene blue treatment; ID50: 50% inhibitory dilution ; N CT: nucleocapsid C-terminal region; N FL: nucleocapsid full protein; RBD: receptor binding domain; S: spike full protein; S2: S2 fragment

Table S5: Ratios of IgA/IgG and cytophilic over non-cytophilic IgG subclasses (IgG1+IgG3 / IgG2 + IgG4) for each of the antigens (N CT, N FL, RBD, S, S2) in study participants at day 7 according to treatment group (placebo and MB-CCP groups) and in CCP units before and after MB-treatment

**Participants**							
**Ratios**	**Ag**	**Overall**	**MB-CCP group**	**Placebo group**	**Placebo - MB-CCP**	***P* value**	**p.sign**
Ratio IgA/IgG	N CT	0.203 (0.712)	0.295 (1.309)	0.145 (0.294)	-0.15	0.013	*
	N FL	0.671 (0.802)	0.568 (0.816)	0.681 (0.779)	0.113	0.386	ns
	RBD	0.5 (1.1)	0.261 (0.797)	0.872 (1.276)	0.611	<0.001	***
	S	0.371 (0.703)	0.222 (0.513)	0.631 (0.993)	0.409	0.000	***
	S2	0.42 (0.437)	0.423 (0.407)	0.409 (0.45)	-0.014	0.631	ns
Ratio IgG1+IgG3 / IgG2+IgG4	N CT	0.976 (5.581)	1.171 (5.11)	0.628 (5.321)	-0.543	0.313	ns
	N FL	14.543 (30.538)	22.533 (27.46)	8.201 (31.304)	-14.332	0.043	*
	RBD	3.167 (18.781)	4.82 (27.862)	1.731 (5.258)	-3.089	<0.001	***
	S	15.729 (37.401)	23.086 (37.559)	8.78 (27.613)	-14.306	0.003	**
	S2	47.643 (72.787)	49.55 (69.692)	45.418 (72.413)	-4.132	0.949	ns
**CCP units**							
**Ratios**	**Antigens**	**Overall**	**After MB**	**Before MB**	**After – Before MB**	***P* value**	**p.sign**
Ratio IgA/IgG	N CT	0.043 (0.058)	0.043 (0.055)	0.045 (0.062)	-0.002	0.875	ns
	N FL	0.032 (0.057)	0.027 (0.058)	0.036 (0.063)	-0.009	0.005	**
	RBD	0.057 (0.095)	0.044 (0.091)	0.07 (0.13)	-0.026	0.078	ns
	S	0.091 (0.144)	0.075 (0.109)	0.101 (0.191)	-0.026	0.005	**
	S2	0.19 (0.226)	0.171 (0.186)	0.25 (0.271)	-0.079	<0.001	***
Ratio IgG1+IgG3 / IgG2+IgG4	N CT	4.588 (8.253)	3.679 (6.524)	6.042 (8.58)	-2.363	0.050	ns
	N FL	31.677 (31.013)	32.232 (31.892)	30.747 (32.048)	1.485	0.198	ns
	RBD	93.078 (89.822)	76.133 (57.272)	129.42 (99.532)	-53.287	<0.001	***
	S	153.748 (148.425)	122.629 (86.379)	231.98 (174.529)	-109.351	<0.001	***
	S2	100.772 (119.725)	100.404 (107.783)	101.502 (132.009)	-1.098	0.198	ns

Ag: antigens; Ig: Immunoglobulin; N CT: nucleocapsid C-terminal region; N FL: nucleocapsid full protein; RBD: receptor binding domain; S: spike full protein; S2: S2 fragment; ns: non-significant; MB: methylene blue
